# The fate of the duplicated androgen receptor in fishes: a late neofunctionalization event?

**DOI:** 10.1186/1471-2148-8-336

**Published:** 2008-12-18

**Authors:** Véronique Douard, Frédéric Brunet, Bastien Boussau, Isabelle Ahrens-Fath, Virginie Vlaeminck-Guillem, Bernard Haendler, Vincent Laudet, Yann Guiguen

**Affiliations:** 1 INRA-SCRIBE IFR 140, Campus de Beaulieu, 35042 Rennes Cedex, France; 2 Institut de Génomique Fonctionnelle de Lyon, Université de Lyon, UMR 5242 du CNRS, INRA, IFR128 BioSciences Lyon-Gerland, Ecole Normale Supérieure de Lyon, 46, Allée d'Italie, 69364 Lyon Cedex 07, France; 3 Biométrie et Biologie Évolutive UMR CNRS 5558 Université Claude Bernard-Lyon 1, 43, Boulevard du 11 novembre 1918, 69622 Villeurbanne Cedex, France; 4 Bayer Schering Pharma AG, 13342 Berlin, Germany

## Abstract

**Background:**

Based on the observation of an increased number of paralogous genes in teleost fishes compared with other vertebrates and on the conserved synteny between duplicated copies, it has been shown that a whole genome duplication (WGD) occurred during the evolution of Actinopterygian fish. Comparative phylogenetic dating of this duplication event suggests that it occurred early on, specifically in teleosts. It has been proposed that this event might have facilitated the evolutionary radiation and the phenotypic diversification of the teleost fish, notably by allowing the sub- or neo-functionalization of many duplicated genes.

**Results:**

In this paper, we studied in a wide range of Actinopterygians the duplication and fate of the androgen receptor (AR, NR3C4), a nuclear receptor known to play a key role in sex-determination in vertebrates. The pattern of AR gene duplication is consistent with an early WGD event: it has been duplicated into two genes AR-A and AR-B after the split of the Acipenseriformes from the lineage leading to teleost fish but before the divergence of Osteoglossiformes. Genomic and syntenic analyses in addition to lack of PCR amplification show that one of the duplicated copies, AR-B, was lost in several basal Clupeocephala such as Cypriniformes (including the model species zebrafish), Siluriformes, Characiformes and Salmoniformes. Interestingly, we also found that, in basal teleost fish (Osteoglossiformes and Anguilliformes), the two copies remain very similar, whereas, specifically in Percomorphs, one of the copies, AR-B, has accumulated substitutions in both the ligand binding domain (LBD) and the DNA binding domain (DBD).

**Conclusion:**

The comparison of the mutations present in these divergent AR-B with those known in human to be implicated in complete, partial or mild androgen insensitivity syndrome suggests that the existence of two distinct AR duplicates may be correlated to specific functional differences that may be connected to the well-known plasticity of sex determination in fish. This suggests that three specific events have shaped the present diversity of ARs in Actinopterygians: (i) early WGD, (ii) parallel loss of one duplicate in several lineages and (iii) putative neofunctionalization of the same duplicate in percomorphs, which occurred a long time after the WGD.

## Background

Actinopterygian fishes have provided the first clear demonstration of an ancient whole genome duplication (WGD) in vertebrate evolution [1]. This event was originally suggested based on the finding that zebrafish and medaka possess seven Hox clusters [2-4], compared to four in mammals and one in most invertebrates. It was confirmed later on by comparative mapping [5] and through the analysis of genome sequences of two pufferfishes [1,6]. Indeed, many short duplicated groups of linked genes were identified in the *Takifugu rubripes *and *Tetraodon nigroviridis *genomes [1,7]. The duplication event leading to these duplicates was dated by molecular clock to a window between divergence of Actinopterygians from Tetrapods, and diversification of teleost fish [8,9]. In addition, all chromosomes of *Tetraodon nigroviridis *were assigned to syntenic groups of duplicated genes, demonstrating the genomic scale of the duplication. It was further shown that each pair of duplicated genes was homologous to one non-duplicated human chromosomal region [1].

**Figure 1:**
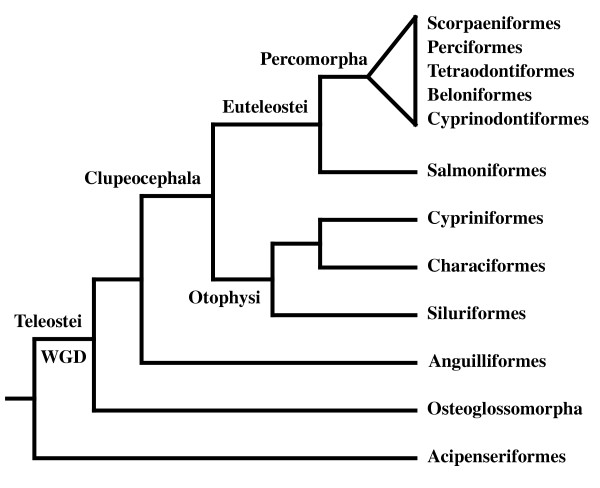
**Classification of the fish species used in this survey as in **[51,52]. The Whole Genome Duplication (also named 3R) event is indicated.

Direct dating of fish gene duplications based on molecular clock was hampered by saturation of synonymous changes at the time scales considered, as well as by differences in evolutionary rates between mammals and fishes [1,10-13]. Less sensitive to these problems, phylogenies of a few tens of gene families have shown a high frequency of gene duplications to be a common feature among sampled teleosts or euteleosts, but not among other fishes [11,14]. Comparative mapping has recently shown that paralogons are homologous between pufferfishes (which belong to Percomorphs) and zebrafish (a Cypriniform), implying that the whole genome duplication event occurred before the divergence of these two lineages of Euteleosts [7]. In addition, using a small number of genes, Hoegg *et al*. [14] have scrutinized the existence of duplications in basal Actinopterygians and have found that the WGD event took place after the split of the Acipenseriformes from the lineage leading to teleosts but before the divergence of Osteoglossiformes, making it specific to the teleostean fish (Figure 1). Because this event separates the species-poor basal lineages from the species-rich teleost lineages, the same authors have suggested that the additional number of genes resulting from this event might have facilitated the evolutionary radiation and the phenotypic diversification of teleosts [15,16].

One basic question regarding gene duplication is the fate of duplicated genes. According to the Duplication – Degeneration – Complementation (DDC) model proposed by Force *et al*. [17], duplicated genes may have three main fates: the majority of duplicated copies are lost, some duplicated genes are subfunctionalized (*i.e*. they share the ancestral function of their non duplicated ancestor) and some others undergo neofunctionalization (*i.e*. they change their function when compared to their ancestor). In most cases the sub- or neofunctionalization events are classically considered to have occurred relatively soon after the duplication event. We recently suggested that a biased subset of genes was retained as duplicates after the genome duplication and that gene retention was biased with regard to biological processes [18]. Most notably, we observed an enrichment of fish genomes in new paralogs implicated in development, supporting the link between genome duplication and fish morphological diversity [15,18]. In addition, several studies have shown that sub- or neofunctionalization events can be observed at the expression level when specific pairs of duplicated genes are studied (see for example [19-21]). Of note, if there are some examples of neofunctionalization events affecting the biochemical function of genes after the vertebrate WGD, much less numerous specific examples of that sort were shown for fish duplicated genes [22-24].

Androgens play essential roles in sex differentiation, sex maturation and behavior in vertebrates, including Actinopterygian fishes [25,26], and their actions are mediated through a specific receptor, AR (NR3C4) which belongs to the nuclear receptor super-family [27]. In Actinopterygians, the mediation of androgen action is far more complicated than in other vertebrates as there is a duality in the active androgens involved in reproduction, *i.e*. regular androgens (DHT) *versus *11-oxygenated androgens (11KT) [26]. Like every nuclear receptor, ARs are composed of three main domains [27]: a hypervariable N-terminal domain involved in transcriptional activation, a DNA-binding domain (also referred to as the C Domain) which permits the binding of receptor on target genes and a ligand-binding domain (also referred to as the E Domain). The amino acid sequences of these last two domains (DBD and LBD) are highly conserved from actinopterygians to mammals with 90% and 70% identity with mammalian ARs for the DBD and the LBD respectively [28]. In Actinopterygians, several duplicates of AR were characterized in the rainbow trout, *Oncorhynchus mykiss *[29], the mosquitofish,* Gambusia affinis *[30], *Astatotilapia burtoni *(see Table 1 for accession numbers), the Nile tilapia, *Oreochromis niloticus*, the Japanese eel, *Anguilla japonica *[31,32] and in *Gasterosteus aculeatus *[33]. Interestingly, in addition to these molecular clones corresponding to two duplicated genes, different forms of ARs, termed AR-A and AR-B, have also been biochemically characterized based on their binding affinities for different androgen ligands in the Atlantic croaker, *Micropogonias undulatus *[34] and the kelp bass, *Paralabrax clathratus *[35]. These two receptors display different tissue distributions with AR-A present only in the brain and AR-B found both in the brain and the gonads [34]. In addition, the different binding affinities of these two ARs suggest that the receptors mediate the actions of different androgens, 11KT and DHT, in different tissues of teleost fish [36].

**Table 1:** Androgen receptors, species names and their accession numbers.

Genus_AR	Scientific name	Common name	Class_Infraclass	Order	SWP_TrEMBL_ID	Ensembl_Prot_ID	GenBank_Acc_Nb
Homo_AR	Homo sapiens	Human	Mammalia	Primates	ANDR_HUMAN	ENSP00000363822	P10275

Mus_AR	Mus musculus	Mouse	Mammalia	Rodentia	ANDR_MOUSE	ENSMUSP00000052648	NP_038504

Gallus_AR	Gallus gallus	Chicken	Aves	Galliformes	Q2ACE0_CHICK	ENSGALP00000007301	BAE80463

Xenopus_t_AR	Xenopus tropicalis	European clawed frog	Amphibia	Anura	n.a.	ENSXETP00000011091	n.a.

Xenopus_l_AR	Xenopus laevis	African clawed frog	Amphibia	Anura	P70048_XENLA	_	AAC97386

Protopterus_AR	Protopterus annectens	Protopterus annectens	Sarcopterygii/dipnoi	Lepidosireniformes	A3QQ74_PROAN	_	ABF50783*

Myoxocephalus_AR-B	Myoxocephalus scorpius	Shorthorn sculpin	Actinopterygii/Teleostei	Scorpaeniformes	A3QQ70_MYOSC	_	ABF50779*

Takifugu_AR-A	Takifugu rubripes	Fugu	Actinopterygii/Teleostei	Tetraodontiformes	n.a.	SINFRUP00000156318*	n.a.

Takifugu_AR-B					n.a.	SINFRUP00000135072*	n.a.

Tetraodon_AR-A	Tetraodon nigroviridis	Spotted green pufferfish	Actinopterygii/Teleostei	Tetraodontiformes	Q4S8Q7_TETNG	GSTENP00022234001	CAG02975

Tetraodon_AR-B					Q4RT97_TETNG	GSTENP00029350001	CAG08385

Tetraodon_f_AR-A	Tetraodon fluviatilis	Green pufferfish	Actinopterygii/Teleostei	Tetraodontiformes	A3QQ72_TETFL	_	ABF50781*

Porichthys_AR-A	Porichthys notatus	Plainfin midshipman	Actinopterygii/Teleostei	Batrachoidiformes	Q4F6Z1_PORNO	_	AAZ14095

Oryzias_AR-A	Oryzias latipes	Medaka	Actinopterygii/Teleostei	Beloniformes	Q76LM5_ORYLA	ENSORLP00000011941*	BAC98301

Oryzias_AR-B					A8CMD5_ORYLA	ENSORLP00000010323*	ABV55993

Rivulus_AR-A	Rivulus marmoratus	Mangrove rivulus	Actinopterygii/Teleostei	Cyprinodontiformes	Q15HT9_RIVMA	_	ABC68612

Gambusia_AR-A	Gambusia affinis	Western mosquitofish	Actinopterygii/Teleostei	Cyprinodontiformes	Q5NU07_GAMAF	_	BAD81046

Gambusia_AR-B					Q5NU08_GAMAF	_	BAD81045

Gasterosteus_AR-A	Gasterosteus aculeatus	Three-spined stickleback	Actinopterygii/Teleostei	Gasterosteiformes	Q801Z2_GASAC	ENSGACP00000026869	AAO83572

Gasterosteus_AR-B					n.a.	ENSGACP00000024489*	n.a.

Micropogonias_AR-A	Micropogonias undulatus	Atlantic croaker	Actinopterygii/Teleostei	Perciformes	Q66VR6_MICUN	_	AAU09477

Acanthopagrus_AR-A	Acanthopagrus schlegeli	Black porgy	Actinopterygii/Teleostei	Perciformes	Q800S7_ACASC	_	AAO61694

Haplochromis_AR-A	Haplochromis burtoni	Burton's mouthbrooder	Actinopterygii/Teleostei	Perciformes	Q8QFV7_HAPBU	_	AAL92878

Haplochromis_AR-B					Q9W6F4_HAPBU	_	AAD25074

Dicentrarchus_AR-A	Dicentrarchus labrax	European sea bass	Actinopterygii/Teleostei	Perciformes	Q4G497_DICLA	_	AAT76433

Dicentrarchus_AR-B					A3QQ67_DICLA	_	ABF50776*

Lepomis_AR-A	Lepomis gibbosus	Lepomis gibbosus	Actinopterygii/Teleostei	Perciformes	A3QQ57_9PERO	_	ABF50766*

Oreochromis_AR-A	Oreochromis niloticus	Nile tilapia	Actinopterygii/Teleostei	Perciformes	Q8UWB7_ORENI	_	BAB20082

Oreochromis_AR-B					Q8UWB8_ORENI	_	BAB20081

Perca_AR-A	Perca fluviatilis	Perch	Actinopterygii/Teleostei	Perciformes	A3QQ55_PERFL	_	ABF50764*

Pagrus_AR-A	Pagrus major	Red sea bream	Actinopterygii/Teleostei	Perciformes	O93497_PAGMA	_	BAA33451

Pomatoschistus_AR-B	Pomatoschistus minutus	Sand goby	Actinopterygii/Teleostei	Perciformes	A3QQ69_POMMI	_	ABF50778*

Halichoeres_AR-A	Halichoeres trimaculatus	Three-spot wrasse	Actinopterygii/Teleostei	Perciformes	Q9DDJ4_HALTR	_	AAG48340

Salmo_AR-A	Salmo salar	Atlantic salmon	Actinopterygii/Teleostei	Salmoniformes	Q8UWF7_SALSA	_	AAL29928

Oncorhynchus_AR-A1	Oncorhynchus mykiss	Rainbow trout	Actinopterygii/Teleostei	Salmoniformes	O93244_ONCMY	_	BAA32784

Oncorhynchus_AR-A2					O93245_ONCMY	_	BAA32785

Cyprinus_AR-A	Cyprinus carpio	Common carp	Actinopterygii/Teleostei	Cypriniformes	A3QQ59_CYPCA	_	ABF50768*

Carassius_AR-A	Carassius auratus	Goldfish	Actinopterygii/Teleostei	Cypriniformes	Q8QFV2_CARAU	_	AAM09278

Ctenopharyngodon_AR-A	Ctenopharyngodon idella	Grass carp	Actinopterygii/Teleostei	Cypriniformes	A3QQ60_CTEID	_	ABF50769*

Labeo_AR-A	Labeo rohita	Indian major carp	Actinopterygii/Teleostei	Cypriniformes	A3QQ58_LABRO	_	ABF50767*

Pimephales_AR-A	Pimephales promelas	Fathead minnow	Actinopterygii/Teleostei	Cypriniformes	Q9I8F5_9TELE	_	AAF88138

Danio_AR-A	Danio rerio	Zebrafish	Actinopterygii/Teleostei	Cypriniformes	A4GVF3_DANRE	ENSDARP00000016299	ABO21344

Gymnocorymbus_AR-A	Gymnocorymbus ternetzi	Black Widow tetra	Actinopterygii/Teleostei	Characiformes	A3QQ71_9TELE	_	ABF50780*

Clarias_AR-A	Clarias gariepinus	Sharptooth catfish	Actinopterygii/Teleostei	Siluriformes	A3QQ63_CLAGA	_	ABF50772*

Heterotis_AR-A	Heterotis niloticus	Heterotis	Actinopterygii/Teleostei	Osteoglossiformes	A3QQ64_9TELE	_	ABF50773*

Heterotis_AR-B					A3QQ65_9TELE	_	ABF50774*

Anguilla_AR-A	Anguilla japonica	Japanese eel	Actinopterygii/Teleostei	Anguilliforme	Q9PWG5_ANGJA	_	BAA83805

Anguilla_AR-B					Q9YGV9_ANGJA	_	BAA75464

Acipenser_AR	Acipenser baerii	Siberian sturgeon	Actinopterygii	Acipenseriformes	A3QQ77_ACIBE	_	ABF50786*

Squalus_AR	Squalus acanthias	Spiny dogfish	Chondrichthyes	Squaliformes	Q56VU2_SQUAC	_	AAP55843

Ginglymostoma_AR	Ginglymostoma cirratum	Nurse shark	Chondrichthyes	Orectolobiformes	A3QQ75_GINCI	_	ABF50784*

Leucoraja_AR	Leucoraja erinacea	Little skate	Chondrichthyes	Rajiformes	Q1KXY2_RAJER	_	ABD46746

Names of the species used in this analysis and their accession numbers retrieved from SwissProt, TrEMBL, Ensembl and Genbank. Asterisks show the genes sequenced in this survey. Non available: n.a.

The case of AR in fish is particularly interesting to study as sex determination mechanisms are known to be particularly plastic in Actinopterygians [37,38]. For example, sequential hermaphroditism is common among marine fishes, particularly in tropical and subtropical seas, and can involve females becoming males (protogyny) or males becoming females (protandry), and also bidirectional (repetitive) sex changes [39-42]. Sex changes among species with well organized social and mating systems are controlled by social cues [41,43-45] and involve complete alterations in gonadal anatomy and function, as well as changes in color and behavior. It is known that sex steroid hormones play important roles in sex change and behavior in many fish species, and androgens have been shown to be crucial for completion of this process in many protogynous hermaphrodites [46-48].

Interestingly, in human, mutations of the AR gene represent the molecular basis of androgen insensitivity syndrome (AIS) [49]. AIS is characterized by defective virilization in 46, XY individuals. The phenotypic spectrum of AIS is extremely large: Complete AIS (CAIS) is characterized by completely female external genitalia. In Partial AIS (PAIS) the phenotype ranges from almost female external genitalia through ambiguous forms to predominantly male external genitalia with hypospadias. Minimal (or Mild) forms of AIS exist which are characterized by impaired spermatogenesis with or without a slight virilization deficit. In addition, the androgen receptor is also implicated in prostate cancer and a specific set of mutations often occurred in patients whose cancer became androgen-independent, an evolution of poor clinical prognosis [50].

In this paper, we reconstructed the evolutionary history of AR in Actinopterygians. We observed a complex history shaped by three successive events well separated in time: (i) an ancestral duplication event specific to teleost fishes corresponding to the WGD; (ii) a parallel loss of one duplicated copy (AR-B) in basal Clupeocephala and (iii) a major sequence divergence indicative of a change in functional constraints in the AR-B duplicate of Percomorphs. This evolutionary history together with the striking mutation patterns is indicative of a putative neofunctionalization event that took place late during AR-B evolution It is tempting to link this neofunctionalization event to the plasticity of sex determination in Percomorphs.

## Results and discussion

### The Androgen receptor is duplicated in teleost fishes

Using a combination of RT-PCR with degenerate primers designed in the conserved C and E domains and *in silico *search against various databases, we were able to characterize 26 different new AR cDNA fragments from 20 different fish species. Along with these AR cDNA fragments, we also identified other steroid receptors (NR3C group) in *A*. *baerii *and *E. stoutii *(Genbank accession numbers ABF50787 and ABF50785). This is probably due to a combination of the high conservation of the C and E domains used to design primers among all steroid nuclear receptors and the low stringency of touchdown PCR procedure that we used. In this study, five new sequences of AR were also identified using database searches and 21 new sequences of AR were isolated by RT-PCR with degenerated primers, the majority of them being teleosteans (20), one being chondrichthyan, one being Dipnoi and one being chondrostean (Table 1).

Of note, one expressed sequence tag in *Oryzias latipes *was found to match with the 5' end region of the divergent AR in *Haplochromis burtoni *(AF121257). The corresponding clone was further sequenced and was confirmed to be a medaka AR. No more than one gene was identified in zebrafish by searching both EST databases and the whole genome sequence (see below). Additional file 1 provides an amino acid alignment of a representative choice of these sequences, focusing on complete DBD and LBD sequences. A complete alignment is available upon request to F.B.

**Figure 2:**
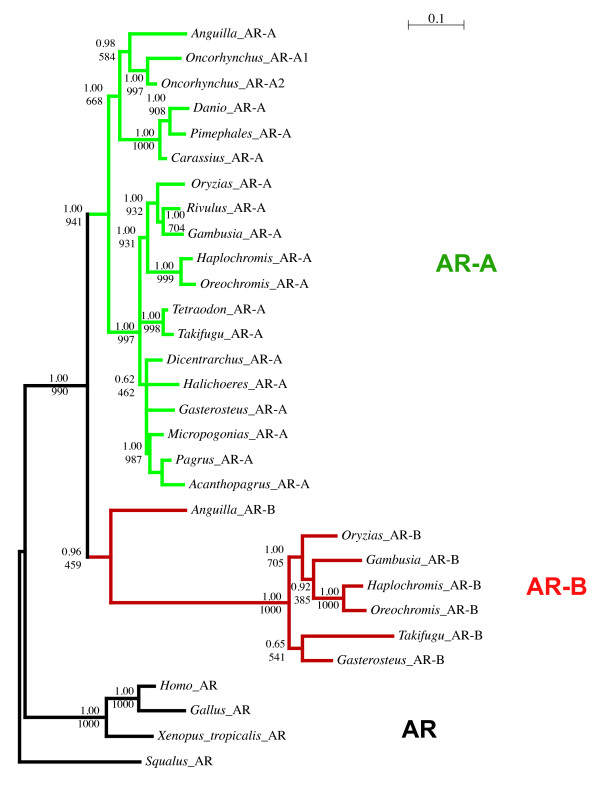
**Phylogenetic analysis of the AR in fish**. Top values are posterior probabilities given with MrBayes. Bottom values are maximum likelihood for 1000 iterations of bootstrap values. Default parameters of PhyML were used. AR-A branches are in green, AR-B branches are in red.

Using the 30 full length sequences identified in our screens, we reconstructed the phylogeny of the Actinopterygian ARs using Neighbor-Joining, Maximum Parsimony, Maximum Likelihood and Bayesian methods (Figure 2 and see Additional file 2). The shorter PCR fragments were used for sequence signature analysis to assess their orthology relationships. Note that given the overall strong conservation of the AR sequences and the relatively short size of the alignable conserved regions, we obtained many branches supported by relatively weak bootstrap values. The AR gene is thus certainly not an adequate marker to decipher fish phylogeny and the topologies we obtained are often not in accordance with accepted concepts in fish phylogeny [51,52]. Nevertheless, the main branches discussed in this paper are well supported and allow the drawing of clear conclusions.

**Figure 3:**
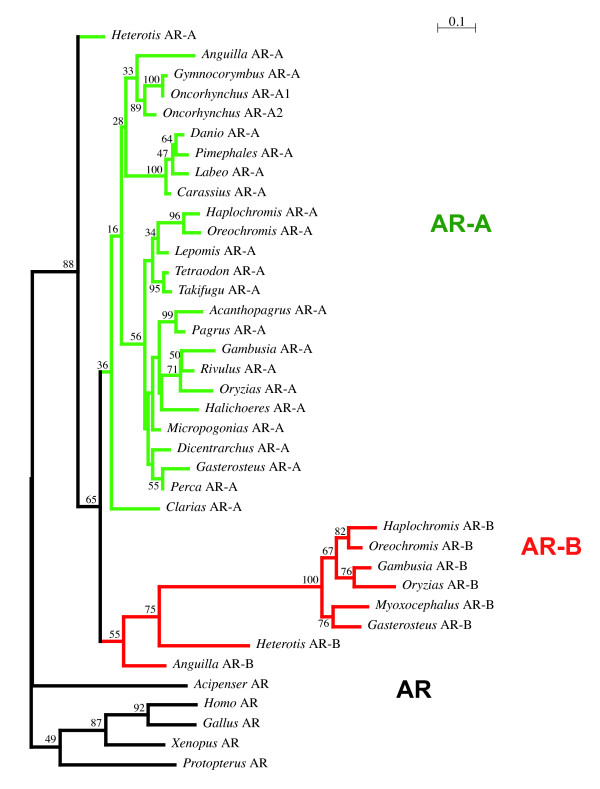
**Maximum likelihood analysis of all species using PhyML**. Default parameters of PhyML were used. 100 bootstrap replicates were used and only values above 30 are indicated. Since short sequences are included here, the bootstrap support is lower than on the tree based on complete sequences as in Figure 2. AR-A branches are in green, AR-B branches are in red.

In all cases, only one AR sequence was identified in each tetrapod. Based on partial sequence analysis, the AR from the west African lungfish branched at the root of this tetrapod cluster (not shown). We obtained only one AR sequence in the sturgeon, *Acipenser baerii *and this sequence is, as expected, clearly located at the base of the Actinopterygian sequences, suggesting that it corresponds to a non-duplicated version of the AR gene (Figure 3). In contrast in *Heterodontis niloticus*, a member of the Osteoglossiformes, which is located at the base of the teleosts according to recent phylogenies based on mitochondrial and nuclear genes [51,53], we found two AR sequences, as in many other teleost fishes such as the eel, the medaka or the cichlids (Table 1 and Figure 2). This suggests that the duplication giving rise to the AR-A and AR-B genes occurred specifically at the base of the teleost tree after the split of the Acipenseriformes from the lineage leading to teleosts but before the divergence of Osteoglossiformes (see Figure 1). This phylogenetic dating obtained with AR is in accordance with the data recently obtained with other genes such as Sox11 and tyrosinase by Hoegg *et al*., [15]. In contrast to the data from these authors, our data set does not contain sequences from Semionotiformes (gars) or Amiiformes (the bowfins, *Amia calva*) that would allow us to confirm more accurately that the WGD occurred specifically in teleosts. Nevertheless, our data are fully consistent with this likely scenario.

**Figure 4:**
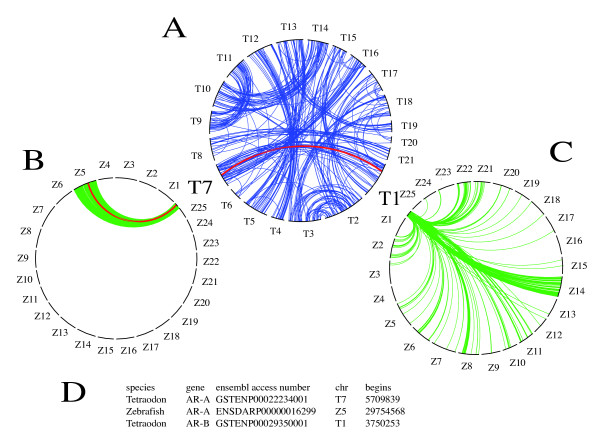
**Syntenic analysis of the AR in fish**. We used the rose window method as described in Jaillon *et al*. [1] and Brunet *et al*. [18] (see also Additional file 3). (A) Rose window showing the 21 Tetraodon chromosomes, illustrating the two-by-two relation between duplicates originating from the teleost specific WGD event. AR-A and AR-B in Tetraodon are located respectively on chromosome T1 and T7, chromosomes that have been described as originating from the teleosts WGD. The red line shows the relationship between these two genes. (B and C) relationship between the genes present in Tetraodon chromosomes T7 (B) and T1 (C) with the genes present in the 25 zebrafish chromosomes. As shown in panel B, the AR A gene is found in the zebrafish on chromosome Z5 (red line) and a very strong synteny exists in general between T7 and Z5 chromosomes. As shown in panel C, a clear synteny is found between T1 and the Z14 chromosomes, although less univocal than in the previous case (orthology based from Ensembl v48, in agreement with reciprocal best-hit analyses we performed, data not shown). Importantly, the AR-B ortholog in the zebrafish could not be detected neither on chromosome Z14 nor on other locations in the whole genome. (D) Table indicating the positions and Ensembl accession numbers of the relevant AR genes.

That the two sequences AR-A and AR-B are indeed the product of the ancestral WGD specific of teleosts is further supported by the chromosomal location of the two tetraodon AR sequences. Indeed, these two genes are located on chromosomes T1 and T7 in the tetraodon genome (Figure 4). From a global synteny analysis, we have previously shown that these two chromosomal regions have a common origin [1,18]. This is further supported by the observation that in the medaka genome, the two AR genes are located on chromosomes M10 and M14 that also share many duplicated genes ([16] and data not shown). Taken together, these data unambiguously show that the AR gene was duplicated to give rise to two paralogs AR-A and AR-B during the teleost specific WGD.

### AR-B was secondarily lost in basal Clupeocephala, including zebrafish

When analyzing the tree presented in Figure 1, we were puzzled to observe that the AR-B sequence can be found in basal teleosts (*Heterotis*, *Anguilla*) as well as in many Percomorphs but is missing in many basal Clupeocephala lineages.

As this observation could be due to an experimental bias linked to a failure to amplify a divergent gene by PCR, we then first checked that this observation was not due to an artifactual lack of detection of the AR-B gene. To this end, we focused our analysis on zebrafish for which a large number of data (complete genome, ESTs, *etc*...) are available. First, we carried out RT-PCR experiments using different batches of primers and several RNA extracts of zebrafish embryos at various developmental stages, as well as adult organs. In all cases, we detected only one AR sequence whereas our primer batches were able to detect divergent NR3C steroid receptors such as GR, MR or PR. PCR experiments based on DNA amplification of short fragments contained in only one exon also failed. Finally, we intensively screened the release Zv7 (13 July 2007) of the zebrafish genome using various fragments of the AR gene as baits without any significant hit. Of note, no sequence reminiscent of a pseudogene was detected.

Due to the availability of a complete and assembled zebrafish genome sequence, we tried to better understand the fate of the AR-B gene in zebrafish. In *Tetraodon*, we found two AR genes, AR-A and AR-B (Table 1 and Figure 2). Since we and others previously showed that an extensive synteny persists between Tetraodontiformes and zebrafish genomes [1,7,54], we precisely mapped in *Tetraodon *and zebrafish the syntenic regions containing AR-A and AR-B. Figure 4 clearly shows that AR genes map in a large duplicated region corresponding to chromosomes T1 and T7 in *Tetraodon*. Chromosome T7 in *Tetraodon *is syntenic to chromosomes Z5, Z10 and Z21 in zebrafish. Interestingly, the zebrafish AR-A ortholog is present in chromosome Z5, as predicted based on conserved synteny. A detailed map shows that the organization of this region is conserved between tetraodon and zebrafish (data not shown). The *Tetraodon *AR-B gene map to chromosome T1 and the region encompassing the gene corresponds mainly to the zebrafish chromosome Z14. The mapping of the region containing the *Tetraodon *AR-B sequence on the zebrafish genome shows that this region has been scrambled during evolution. Many gene orders are not conserved and large fragments are missing or were exchanged (data not shown). The same consideration is reached when the medaka genome is considered (see Additional file 3).

These data indicate clearly that a secondary loss of AR-B occurred in zebrafish. Interestingly, in related Cypriniformes (5 species), Characiformes (1 species) and Siluriformes (1 species) that altogether form the well-supported clade Otophysi [55], we also found only one AR-A sequence and no AR-B one. We recently screened EST data available for all these species and we could not find any sequence reminiscent of AR-B. Of course, although complete genome sequences are not available and RT-PCR results can artifactually miss a divergent sequence, these data collectively suggest that an unique event of loss of AR-B occurred early on in the Otophysi lineage.

**Figure 5:**
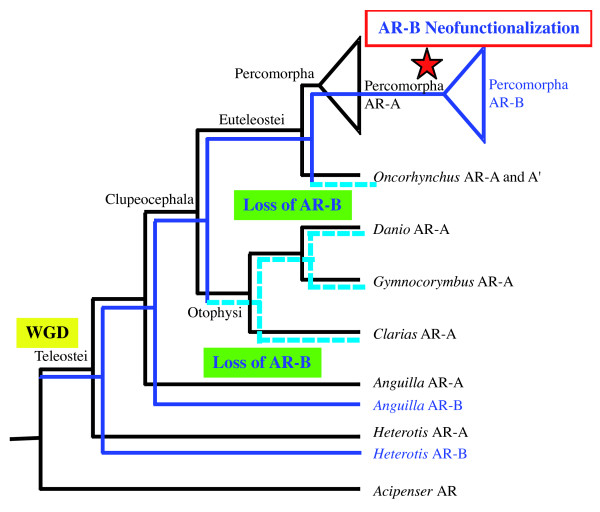
**Model of evolution of the AR in fish based on the same phylogenetic background as Figure **1. A WGD occurred at the base of the Teleostei, and two successive losses occurred in the lineages leading to the Otophysi and the Oncorhynchus. Last, in the percomorphs, all the AR-B genes harbor an event of acceleration, while the AR-A ones show no sign of such rapid evolution at the molecular level.

Strikingly, the exact same situation was observed in another independent lineage of Clupeomorph: the Salmoniformes. In the 2 species analyzed one more time, we found only AR-A and not AR-B. Of note, in Salmoniformes two AR-A sequences are observed (corresponding to the two subtypes named *ar-alpha *and *ar-beta *known in rainbow trout [29]). These correspond to the tetraploidization event that occurred 25 to 100 MYr ago specifically in the salmonid lineage [56]). It is important to note that even in the divergent AR-B sequences that we analyzed in Percomorphs (see below), the regions targeted by the various PCR primers that we used are well-conserved. In addition, once again, the screening of the ESTs available in salmon and trout (the most widely used species of Salmoniformes in aquaculture and genomic research) has not delivered any AR-B type sequence. Thus, although this conclusion is only tentative in the absence of a complete genome, the most likely scenario, considering all existing data, is that AR-B gene was lost in Salmoniformes as in Otophysi (Figure 5).

It is clear that loss of duplicated genes is a very common fate after a genome duplication event but the present analysis nicely illustrates a late case of neofunctionalization. Our data suggest that basal teleosts and percomorphs kept two functional copies of AR whereas "intermediate" lineages such as Otophysi and Salmoniformes lost it secondarily (see below). According to the topology of teleost fish phylogeny presented in Figure 1, our results imply two independent losses of AR-B, one at the base of Otophysi and one at the base of Salmoniformes (see also Figure 5). This is based on the assumption that the current topology based essentially on complete mitochondrial DNA analysis is correct in the respective placement of Salmoniformes and Otophysi [51,55,57]. If, as suggested by some authors, these two groups form a monophyletic clade, it may be possible that in fact only one ancestral event of loss occurred [58]. In that case, we can predict that AR-B should not be found in Esociformes. In any case, our present data plead for the search of AR-A and AR-B in orders of Actinopterygians located at key positions in the evolutionary tree: it would be interesting for example to see if AR-B is present in other Ostariophysi lineages such as Gonorhynchiformes, or Clupeomorphs [55] as well as other Protacanthopterygii such as Esociformes, Argentinoidea and Osmeroidea. This will allow a more precise determination of when the events of loss occurred [51].

It is difficult to speculate with the data available why the Otophysi and the salmonids apparently do not need a second AR-B gene. Given the major function of AR in sex determination and sex organ differentiation, it is tempting to link these events with these processes but given that these data on Otophysi and salmonids are limited to some specific models such as zebrafish, salmon and trout, it is up to now difficult to find an obvious connection. It is striking that zebrafish and salmonids are extremely different regarding sex determination and sex differentiation. In addition, as discussed above, the exact phylogenetic range of this loss of AR-B is still unclear.

### Functional shift of AR-B in Euteleosts

In the tree presented in Figure 2, we noticed the presence of a highly divergent terminal group of AR-B sequences. This is confirmed when a larger dataset including partial sequences is used to construct a phylogeny with any of the 4 methods used (Figure 3 and data not shown). In all cases, we found a long terminal branch uniting divergent AR-B sequences. This divergent AR-B subtype unambiguously (bootstrap value: 1000 out of 1000; posterior probabilities: 1.00) clusters AR sequences of fish belonging to the percomorphs, *i.e*., the seabass *D. labrax*, the sand goby, *P. minutus*, the nile tilapia, *O. niloticus *and *A. burtoni*, a scorpaeniforme with *M. scorpius*, the shorthorn sculpin,; a beloniforme with the medaka, *O. latipes*, a cyprinodontiforme with the mosquitofish, *G. affinis*, and 2 tetraodontiformes with the tetraodon, *T. nigroviridis *and the fugu, *T. rubripes*. Indeed, when we considered the sequence alignment (see Additional file 1), we observed a serie of mutations that are present only in the percomorph AR-B sequences (highlighted in green). The divergence of these sequences corresponds to a transient episode of sequence divergence as the AR-B sequences clustered inside this group are not particularly variable. Thus, all these data suggest that the percomorphs AR-B are connected to the basal teleosts AR-B through a long branch and exhibit some striking sequence divergence at key positions. From the phylogenetic range of species in which these divergent AR-B sequences are found, it is likely that this acceleration occurred specifically in percomorphs, although this remains to be fully established by a broader taxonomic sampling including other Neotelestoi lineages such as basal Acanthomorphs (e.g. Gadiformes; [59]) as well as Bericyformes [52]. To really assess if this event is found in all Percomorphs, some basal lineages (e.g. Ophidiiformes) of this extremely vast group of fishes should also be studied [52]. In the mean time, given our observation that divergent AR-Bs are found only in percomorphs from our dataset, we will refer to these divergent sequences as "percomorph AR-B".

It is important to insist on the fact that basal teleosts (*Anguilla *and *Heterotis*) clearly contained AR-A and AR-B paralogs. For AR-A, this is not difficult to establish given that this gene is present in a wide phylogenetic range of species. For AR-B, the assignment is less obvious since, as discussed above, this gene has been lost in basal Clupeocephala. The fact that the AR-Bs from *Anguilla *and *Heterotis *are indeed orthologs of the percomorph AR-B is indicated by several features: (i) these sequences exhibit a few key sequence signatures that represent synapomorphies of AR-B sequences (highlighted in yellow and orange in Additional file 1), this is for example the case of Gly633, Ser861 and Ser928 in the LBD; (ii) the topology of the phylogenetic tree supports this assumption albeit with a moderate support (posterior probability of 0.96, bootstrap value of 459‰; Figure 2). Of note, we constructed trees based on Bayesian analysis which confirm that the topology presented in Figures 2 and 3 is robust (see Additional file 2).

The most likely scenario accounting for the data available concerning Actinopterygian AR evolution is therefore a three step model (Figure 5): (i) ancestral duplication of a unique AR gene during the WGD event specific of teleost fishes. This explains why *Anguilla *and *Heterotis *have two AR sequences, AR-A and AR-B; (ii) secondary loss of AR-B in basal Clupeocephala (Otophysi and Salmoniformes) explaining the restricted phylogenetic occurrence of AR-B when compared to AR-A; (iii) a late specific divergence of AR-B. The long branch connecting percomorph AR-B to the basal AR-B sequences is indicative of the accumulation of numerous mutations and we thus proposed that it corresponds to a functional shift that has affected the AR-B protein.

We therefore wanted to test whether the two groups of paralogous genes AR-A and percomorph AR-B were under different selective pressures. We reasoned that if selective pressures differed between the two groups, there should be sites undergoing substitutions in the AR-A subtree and constrained in the percomorph AR-B subtree, and symmetrically sites constrained in the AR-A subtree undergoing substitutions in the AR-B subtree. Patterns of evolutionary rates in one subtree *versus *the other were compared to answer this question: are they significantly more different than they would be if branches were picked at random among the two subtrees? Expected numbers of substitutions were estimated for all branches of the tree and all sites of the alignment [60]. A non-symmetric correspondence analysis was applied on these numbers of substitutions, and the percentage of variance between branches explained when branches are clustered according to the two subtrees was computed. The significance of this percentage was assessed by a permutation test based on 500 000 replicates, where branches are picked randomly from the two subtrees. Among the 500 000 random clusterings, only 0.36% explained a higher percentage of variance among branches than the clustering according to the paralogous subtrees (See Additional file 4). As branches have been normalized with respect to their lengths by the correspondence analysis, this variance comes from differences in patterns of substitutions, not branch lengths. Therefore, patterns of substitutions are significantly more different between the two groups of paralogous genes than between two random groups of branches. This suggests that selective pressures differ between AR-A and AR-B genes, which is in favor of a possible neofunctionalization. The same conclusion is reached with the use of the PAML software [76]. A significant change in the selective pressure onto the branch specific to the AR-B in percomorphs (p-value = 1.688343e-08) is unequivocally detected, although the test is not sensitive enough to tell whether it is a relaxation of the selective pressure or positive selection that drove this change. It should be made clear that, in the absence of a functional characterization, including a comparison of a basal non-duplicated AR (*e.g*. sturgeon), duplicated AR with a non-divergent AR-B (*e.g*. eel) and duplicated AR with a divergent AR-B (*e.g*. medaka) this neofunctionalization cannot yet be formaly proved and should be regarded as only putative.

This pattern of a late spectacular divergence of a duplicated gene in a precise taxonomic group is an interesting case in which the duplication and the functional shifts are clearly two recognizable events that were decoupled in time. The AR-B gene will thus be a very interesting model to study the precise functional and biological impact of these two events since we have sequences of non-duplicated fish AR (sturgeon), duplicated AR-A (in eel and medaka for example), duplicated and non divergent AR-B (eel) and duplicated and divergent AR-B (medaka). In addition, we have other interesting cases for comparison such as a unique zebrafish AR-A gene with secondary loss of AR-B. The fact that AR is a gene encoding a nuclear hormone receptor with a known ligand, a clear biological role and for which several functional tests are available renders this gene particularly suitable for a precise integrated study of the consequences and respective roles of duplication and evolutionary sequence divergence. For example, it may be very interesting to study if, as proposed recently at a broader scale for nuclear receptors, sequence divergence is correlated to expression divergence [19].

### Analysis of the substitution pattern in relation to human Androgen Insensitivity Syndrome

**Figure 6:**
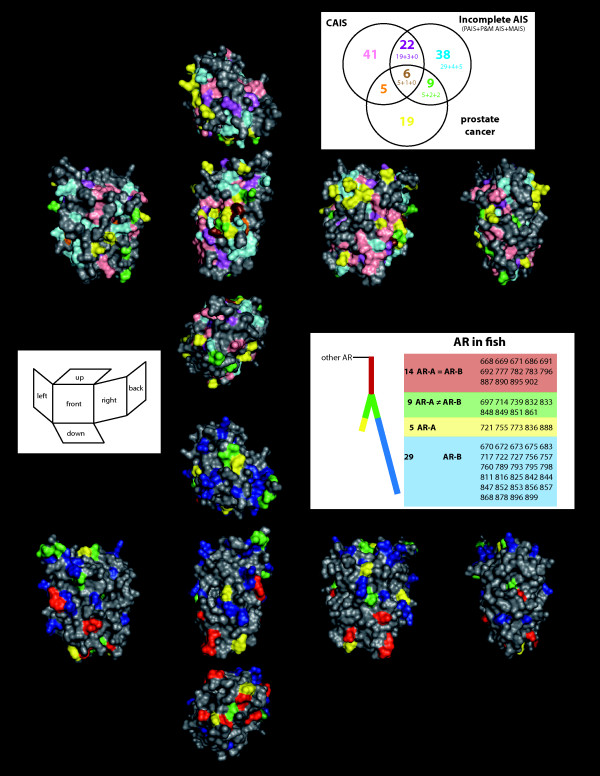
**Location of mutations in the 3D structure of the androgen receptors**. Cubic view of localization of the substitutions onto the surface representation of the LBD of the human AR (1XNN.pdb). (Top): number of substitutions characterizing CAIS, PAIS + MAIS, and the prostate cancer. The mutations are those listed in Additional file 6. Amino acid changes (regardless of the kind of substitutions) can lead to different phenotypes: *e.g*. 5 is the number of aa found in CAIS in a patient, and prostate cancer in another; 9 is the sum of 5 aa reported as found in both PAIS and prostate cancer, etc... See details in Additional file 7. Color codes are reported in the 3D structure. (Bottom): Localization of the substitutions characterizing AR-A and AR-B in fish compared to all other AR. There are 14 identical substitutions in the 2 ARs (that are found in fish AR but not in other vertebrates). We found respectively 14 and 38 substitutions in AR-A and AR-B, 9 of them being common with different substitutions in these two ARs (in green). Color codes are reported onto the 3D representation.

As a first step to analyze the possible consequences of the duplication and divergence of AR-B in Percomorphs, we scrutinized the mutations found in divergent AR-B *versus *AR-A. We first detected how the various mutations observed in Actinopterygian ARs are located in function of the complex evolutionary history described above. We thus categorized the mutations in four classes (Figure 6 and see Additional file 5): (i) mutations found only in AR-A and not in AR-B or ARs from Amniotes (yellow in Figure 6); (ii) mutations observed specifically in divergent AR-B (red star clade in Figure 5; shown in blue in Figure 6); (iii) positions found to be identical in AR-A and AR-B but different in Amniotes AR (red in Figure 6); and finally (iv) positions that are different in Amniotes AR-A and AR-B (green in Figure 6). From this analysis, it is obvious that the divergent AR-B effectively accumulated mutations. Out of 58 amino acid mutations, we found that AR-As exhibit 14 (= 5 + 9; see Figure 6) specific mutations whereas divergent AR-B have 38 (= 29 + 9) specific mutations. This difference is highly significant (Chi-square test ≥ 15.34***).

We thus studied in more detail the 38 mutations found in the divergent AR-B sequences in order to see if some of them could have obvious functional consequences. Of note, and not surprisingly, none of these mutations affect the positions known to directly interact with the ligand or implicated in coactivator binding as determined in the 3D structure of the AR LBD complexed with various ligands [61-63]. Few specific changes are observed such as L744V, M749L, Q783H and M895I (numbering according to the consensual human AR mutations database). Some substitutions are also observed in the AF-2 region: A898 is substituted to a S in most AR-Bs and to a G in most AR-As, as well as I899 is substituted to a V in most AR-Bs. Of note, they are observed in both AR-As and divergent AR-Bs and overall, they are unlikely to account for significant functional consequences.

Then, we scrutinized the positions in the DBD and LBD divergent in AR-B and we checked whether these mutations are affecting amino acids found mutated in human pathologic conditions (Figure 6 and see Additional file 6). We were particularly interested by mutations occurring in Androgen Insensitivity Syndrome (AIS) or prostate cancer since these pathologies affect the ability of the receptor to regulate transcription of target genes in response to ligand binding. AIS is a pathologic condition in humans defined by the eventual occurrence of female differentiation despite the male XY genome and results from germinal mutations in the human AR gene. As discussed above, AIS can be complete (CAIS), partial (PAIS) or mild (MAIS) [49]. We noted that effectively, some divergent AR-B specific mutations are localized in close proximity to functionally relevant residues and may thus impact, in a subtle manner, the function of the receptor. For example the substitution Y739L is observed in all divergent AR-Bs (see Additional file 1). Located close to M742, another amino acid involved in ligand binding, Y739 could influence ligand binding by itself since its substitution to aspartic acid has been described in a CAIS patient [64]. The same question addresses several other amino acids specifically different in divergent AR-Bs as compared to AR-As, such as the F856L substitution which has been observed in patients with CAIS [65] (see Additional file 6).

We thus compared the mutation pattern observed in the divergent percomorphs AR-B gene (that is 38 mutations found in the LBD as well as 22 mutations in the DBD) which are linked to a neofunctionalization event to the mutation pattern occurring in the pathological conditions (Figure 6 and Table 2; see also Additional file 7). When these various types of mutations are mapped on the structure of the human receptor (see Figure 6 for the LBD and Additional file 5 for the DBD), we found that most of the mutations found in AR-B are distinct from pathological mutations. Nevertheless, as discussed above, we also observed some positions mutated both in AR-B and in human patients. Globally, 6 of the 38 LBD mutations found in the divergent AR-B involve amino acids known to be implicated in PAIS + MAIS, whereas only 3 are found in CAIS (see Androgen Receptor Gene Database [75] and references herein; see Additional file 6). Although this difference is not statistically significant, this is in accordance with the notion that AR-B is a functional gene the function of which is only modified (and not drastically reduced) by the specific mutations arising during the putative neofunctionalization event. The fact that we found 7 mutations common between fish AR-B and prostate cancer mutants is more difficult to interpret since mutated ARs in prostate cancer are functionally diverse (from totally inactive receptors to receptors activated by antagonists).

**Table 2:** Common substitutions in fish AR and AR diseases in human.

			CAIS		PAIS+MAIS		prostate cancer	
	DBD	LBD	DBD	LBD	DBD	LBD	DBD	LBD

Length/nb	86	257	14	74	17	75	7	39

AR-A all	2	14	0	1	0	1	0	2

AR-B all	22	38	0	3	0	6	1	7

AR-A = B	2	14	0	1	0	3	0	4

Number of substitutions common to the various human phenotypes of AR diseases and AR-A and AR-B in fish (see Additional file 6 for details)

## Conclusion

The above analyses suggest that these sequence differences between AR-A and AR-B will affect the functionality of these receptors and are linked to a putative neofunctionalization event. This remains of course to be directly addressed through *in vitro *and *in vivo *analysis of the role of AR-A and AR-B in suitable fish models. How this functionality is precisely affected remains therefore an open question. The teleost duality in terms of active androgens involved in reproduction [26] is of great interest in that context. Of note, AR-A and AR-B have been shown to both bind regular androgens and the fish specific 11-oxygenated androgens (11KT) although no direct comparison between AR-A and AR-B has been carried out in the same species until now. We thus have no clear comparison of the respective affinities and potencies of AR-A and AR-B for DHT and 11KT. Of special interest are the *in vivo *binding studies carried out in two perciform species, *i.e*. the Atlantic croaker, *Micropogonias undulates *and the kelp bass, *Paralabrax clathratus*, that demonstrated the existence of two different nuclear androgen receptors that may mediate the physiological actions of different androgens [34-36]. The mutation pattern we observed in AR-B is indicative of a neofunctionalization event at the functional level, but it is likely that this may also be coupled to differences at the expression level. Indeed, neofunctionalization of the expression pattern has been suggested in the cichlid fish *A. burtoni *in which it has been shown that AR-A and AR-B have distinct expression patterns in the brain [66], with a differential implication of these receptors in the maintenance of social dominance status of male fish [67]. Taking into account the functional shift that we specifically observed in the Percomorph lineage, it is tempting to link this functional shift and the sexual lability that is observed in this lineage as the Percomorphs contain nearly 90% of all the hermaphrodite species known to date [37]. One may thus hypothesize that the existence of two functionally divergent AR genes play a role in the plasticity of sex determination often observed in these fishes. In this sense, the presence of the divergent AR-B gene could be viewed as a permissive factor allowing the evolvability of divergent sex determination in these fishes.

## Methods

### Fish and RNA extraction

Common and scientific names of all fish species used in this study are given in Table 1. Tissues samples (whole fish, ovaries, testis and brain) were obtained from fish specimens collected in the wild (*Perca fluviatilis*, *Lepomis gibbosus*, *Pomatoschistus minutus*, *Myoxocephalus scorpius, Protopterus annectens, Labeo rohita *and *Eptatretus stoutii*), bred in captivity in experimental or aquaculture facilities (*Dicentrarchus labrax*, *Ctenopharyngodon idella*, *Gambusia fluvalis, Tetraodon fluvalis*, *Danio rerio*, *Carassius auratus*, *Clarias gariepinus*, and *Heterotis niloticus*) or from local aquarium fish retailers (*Gymnocorymbus ternetzi *and *Acipenser baerii*). For *Ginglymostoma cirratum*, we used the Epigonal Nurse Shark cDNA Library provided by Dr. M.F. Flajnik at the University of Miami School of Medicine [68]. All animals were anesthetized with 2-phenoxyethanol and then sacrificed by decapitation before the sampling of tissues. Total RNA was prepared after homogenization in TRIZOL^® ^reagent (Invitrogen, Cergy Pontoise, France) following the manufacturer's instructions and mRNA was further purified from 500 μg of total RNA using the OligoTex mRNA kit (Qiagen).

### Reverse transcription

For cDNA synthesis, 2 μg of mRNA were denatured in the presence of oligo (dT) (0.5 μg) for 5 min at 70°C, and then chilled on ice. Reverse transcription (RT) was performed at 37°C for 1 hour using M-MLV reverse transcriptase (Promega, Madison, WI) as described by the manufacturer. Namely, 2 μg of mRNA were reverse transcribed with 200 units of M-MLV reverse transcriptase in the presence of 1.25 μl of each dNTP at 10 mM, 5 μl of M-MLV 5× reaction buffer and 25 units of RNasin^® ^(Promega, Madison, WI, USA), in a total volume of 25 μl.

### Primer design

Degenerate oligonucleotide primers were designed after alignment of various fish, reptile, bird and mammalian AR amino acids sequences. Two degenerate primers, AR.AS: TGY TAY GAR GCI GGI ATG AC [CYEAGM] and AR.AAS: AAI ACC ATI ACI BYC CTC CA [WMGVMVF], were selected respectively in the highly conserved DBD (C domain) and steroid LBD (E domain) regions. The alignment of the divergent *H. burtoni *AR-B (see Table 1) sequence was used to design another specific degenerate primer, AR.BS: TGC TTY ATG KCG GGN ATG [CFMSGM] in the same region as AR.AS. AR.AS and AR.BS were both used in conjunction with AR.AAS [AR.AS × AR.AAS and AR.BS × AR.AAS]. A second set of degenerate primers outside the PCR fragments produced by AR.AS × AR.AAS or AR.BS × AR.AAS was designed: AR3S: GTI TTY TTY AAR AGR GCI GC [VFFKRAA] and AR3AS: CCA ICC CAT IGC RAA IAA IAC CAT [MVFAMGW] and were used in a nested PCR strategy.

### Touchdown RT -PCR and cloning of AR sequences

The RT-PCR strategy was used according to Escriva *et al*. [69]. PCR reactions were set up using 2 μl of cDNA or 2 μl of Epigonal Nurse Shark cDNA library and 0.5 units of *Taq *Polymerase (Sigma), 200 μM of dNTPs, 30ρmol of each degenerated primer and 2.5 μl of Taq buffer 10× (Sigma). The total volume of the reaction was 25 μl and the cycling Touchdown PCR conditions were: 94°C for 1 min, 20 cycles of regularly decreasing annealing temperature from 50°C to 40°C for 30 sec and 72°C for 30 sec, and 30 cycles at the annealing temperature of 40°C, ending at 72°C for 30 sec. PCR products were analyzed on agarose gel (1%) and amplified DNA fragments of the anticipated length (average between 370 and 450 bp) were subsequently subcloned into pCR 2.1 plasmid. Bacteria (INVαF', *E. coli *TOP10 cells, Invitrogen) were transformed by electroporation, spread on LB-ampicillin agar plates and incubated overnight at 37°C. From 10 to 20 randomly selected recombinant colonies were then screened either using PCR with primers amplifying the inserts (T7 and M13 reverse primers) or by hybridization of nitrocellulose membrane lifts with a rainbow trout AR radiolabelled (dCTP^32^) probe. Positive clones were sequenced using a dideoxy cycle-sequencing method with the Dye Terminator Cycle Sequencing Kit (Applied Biosystems) and reaction sequences were read on an ABI PRISM 310 Genetic Analyzer (Applied Biosystems). A secondary nested PCR was carried out for RNA samples from *G. ternezi*, *D. rerio*, *P. annectens*, and *A*. *baerii *species (see Table 1) using as template a first PCR reaction, obtained using the primers [AR3S × AR3AS], at a 1/100 dilution and a second set of nested degenerate primers [AR.AS × AR.AAS] or [AR.Bs × AR.AAS]. PCR conditions, subsequent cloning, clone selection, and sequencing were as described above.

### Searching AR in sequence databases

Homologous DNA and protein fish ARs were searched on available public databases (non redundant, Expressed Sequence Tags) using the various BLAST programs available through the network servers at the National Center of Biotechnology Information http://www.ncbi.nlm.nih.gov/BLAST/. We also retrieved AR sequences from the whole genome databases at the Ensembl Genome browser http://www.ensembl.org/index.html. From Ensembl v48 (Aug. 2007), we retrieved AR sequences belonging to the Ensembl family ENSF000000000291.

### Sequence and structural analysis

Multiple alignments of the deduced amino acid sequences were generated with Muscle using the default parameters [70]. Phylogenetic trees were realized by multiple alignments of deduced amino acid sequences using the neighbor-joining and parcimony methods implemented in PhyloWin [71]. PhyML [72] was used to generate maximum likelihood phylogenetic trees. Bayesian trees were generated using MrBayes v3 http://mrbayes.csit.fsu.edu/index.php.

To test whether the two groups of paralogous genes AR-A and AR-B were under different selective pressures, patterns of substitutions estimated in the AR-A subtree *versus *the AR-B one in percomorphs were compared. Expected numbers of substitutions per site and per branch were estimated with the CoMap program [60] based on the Bio++ library [73]. This produced a matrix containing branches of the tree as rows, and sites of the alignment as columns. Branches belonging to the AR-A and AR-B subtrees were selected, discarding the two eel sequences, so that the number of branches was the same in the two subtrees. A non-symmetric correspondence analysis was applied on the resulting submatrix, and the percentage of variance between branches explained when branches are clustered according to the two subtrees computed. The significance of this percentage was assessed by a permutation test based on 500,000 replicates, where branches were clustered randomly. All these analyses were conducted with the ade4 package [74] in the R environment (R development core team).

In order to determine any change in the selective pressure along the branch leading to the percomorphs AR-Bs beside of this previous test, we also used PAML version 4 http://abacus.gene.ucl.ac.uk/software/paml.html[76].

Location of all the substitutions found in the LBD of the human AR was retrieved from Bruce Gottlieb's database at his web site http://androgendb.mcgill.ca/[75]. Amino acid substitutions in this human AR database and those specific to the two main lineages were thus positioned onto the 3D structure using PyMOL (by Warren L. Delano, version 2004, http://pymol.sourceforge.net/).

## Authors' contributions

VD performed most of the experimental work (cloning, sequencing of fish ARs), FB performed the bioinformatic analysis (phylogeny, synteny and structural data), BB studied the selective pressures acting on the ARs, IA and BH contributed to the cloning/sequencing steps, VVG and FB compared the pattern of mutations present in fish AR with those found in human patients, VL and YG designed the study and wrote the paper.

## Supplementary Material

Additional file 1 **ARs sequences alignment.** Alignment of the DBD and LBD sequences of ARs compared to that of human (see Table 1 for accession numbers). In this alignment, a dot refers to the same aa as in the first sequence. Sequences not known in 5' and 3' are shown by a hyphen ("-") sign, as well as gaps. AR-B sequences are visualized by the grey background. Alignment with other non AR sequences are available upon request. In red are the gaps or insertions characterizing the ARs in fish. Amino acids shown in green are those conserved in divergent AR-B sequences whereas those in yellow are the substitutions characterizing all AR-Bs. Positions in blue are those specific to AR-A and those in violet are common to the actinopterygian ARs. Click here for file

Additional file 2 **Phylogenetic analyses of complete AR-A and AR-B.** Phylogenetic analyses of complete AR-A and AR-B with sequences encompassing the DBD and the LBD. Four methods were used: bayesian with MrBayes, maximum likelihood (ML) with PhyML with 1000 bootstrap replicates; maximum parcimony (MP) and neighbor joining (NJ) as implemented in Seaview. Click here for file

Additional file 3 **Orthology relationships between medaka, tetraodon and zebrafish chromosomes.** As referenced in legend Figure 4, these rose windows show the orthology relationship between chromosomes on which AR A and AR-B are located in the medaka, Tetraodon and the zebrafish. The excellent synteny observed between the chromosomes are strong remnants of the WGD that occurred specifically in the Teleost lineage. The red lines show the orthology link of the ARs among all the other orthologs (orthology based from Ensembl v48, in agreement with reciprocal best-hit analyses we performed, data not shown) shown here in green. (A) AR-A is found on chromosome T7 in the Tetraodon and on chromosome M14 in the medaka. A strong synteny is observed between these two chromosomes. (B) AR-B is found on chromosome T1 in the Tetraodon and on chromosome M10 in the medaka. A strong synteny is observed is also observed between these two chromosomes. (C) AR-A is found on chromosome Z5 in the zebrafish and on chromosome M14 in the medaka. An unequivocal synteny is observed between these two chromosomes, as shown in Figure 4-B between this zebrafish chromosome and that of the Tetraodon. (D) Although AR-B is found on chromosome M10 in the medaka and that a good synteny is observed with the chromosome Z14 in zebrafish, as observed in Figure 4-C for this species with the chromosome T1 of Tetraodon, the zebrafish lacks AR-B to the point we could not detect its pseudogene. (E) Table indicating the positions and Ensembl accession numbers of the relevant AR genes. Click here for file

Additional file 4 **Statistical distributions of the AR-A and AR-B substitutions in fish.** Statistical representation of the distributions of the AR-A and AR-B substitutions in fish. The bell distribution is a random distribution of the substitutions, the diamond shows that the specificity of AR-A and AR-B *versus *the other AR is statistically clearly not random. Click here for file

Additional file 5 **Human and fish substitutions along the DBD of the human AR.** Representation of the DBD of the human AR modified from [77]. The aa in green are the ones specific to AR-B; in blue, the ones specific to AR-A; in orange and red, when respectively the aa is hit by a common substitution or a different one when compared to other vertebrate ARs. Arrowheads refer to mutations found in CAIS, PAIS, MAIS and prostate cancer respectively in color pink, orange, blue and black. Different aa substitutions are shown by arrowheads side by side. Click here for file

Additional file 6 **List of human substitutions in AR leading to AIS phenotypes and prostate cancer.** List of substitutions in human AR leading to CAIS, PAIS, MAIS and prostate cancer phenotype, as referenced into Bruce Gottlieb's database (androgendb.mcgill.ca/AR23C.pdf) [75], and list of the AR-A and AR-B specific substitutions detailed in Additional file 1. Click here for file

Additional file 7 **Detailed analysis of Table **2. Click here for file
