# Supplementary material for: The fate of the duplicated androgen receptor in fishes: a late neofunctionalization event?
Source: BMC Evol Biol. 2008 Dec 18;8:336. doi: 10.1186/1471-2148-8-336 (PMC2637867; doi:10.1186/1471-2148-8-336)
Supplement: Additional file 1 — ARs sequences alignment. Alignment of the DBD and LBD sequences of ARs compared to that of human (see Table 1 for accession numbers). In this alignment, a dot refers to the same aa as in the first sequence. Sequences not known in 5' and 3' are shown by a hyphen ("-") sign, as well as gaps. AR-B sequences are visualized by the grey background. Alignment with other non AR sequences are available upon request. In red are the gaps or insertions characterizing the ARs in fish. Amino acids shown in green are those conserved in divergent AR-B sequences whereas those in yellow are the substitutions characterizing all AR-Bs. Positions in blue are those specific to AR-A and those in violet are common to the actinopterygian ARs. [file 1471-2148-8-336-S1.doc]

546

Homo_AR VLPIDYYFPP QKTCLICGDE ASGCHYGALT CGSCKVFFKR AAE-----GK [590]

Gallus_AR I......... .......... .......... .......... ...-----.. [590]

Xenopus_t_AR L......... .......... .......... .......... ...-----.. [590]

Leucoraja_AR -FR.QVW--- ---------- ---------- ---G...... ...-----.. [563]

Squalus_AR G.QM..F.Q. .......... .......... .......... ...VLSLL.. [595]

Ginglymostoma_AR ---------- ---------- ---------- ---------- ---------- [545]

Protopterus_AR ---------- ---------- ---------- ---------- ---------- [545]

Acipenser_AR ---------- ---------- ---------- ---------- ---------- [545]

Takifugu_AR-B ---------- -RV.MV...D ........V. .......... ..A-----.. [579]

Tetraodon_f_AR-B ---------- ---------- ---------- ---------- ---------- [545]

Myoxocephalus_AR-B ---------- ---------- ---------- ---------- ---------- [545]

Pomatoschistus_AR-B ---------- ---------- ---------- ---------- ---------- [545]

Oreochromis_AR-B FF.-GV.LSE RRV.QV...G ........V. .......... ..A-----.. [589]

Haplochromis_AR-B FF.-GV.LSE RRV.QV...D ........V. .......... ..A-----.. [589]

Gasterosteus_AR-B LF.-GV.LSD RRV.QV...D ........V. .......... ..A-----.. [589]

Oryzias_AR-B LF.-GV.LSD RRV.QV...D ........V. .......... ..A-----.. [589]

Gambusia_AR-B FF.-GVFLSD RRV.QV...D ........V. .......... ..A-----.. [589]

Heterotis_AR-B ---------- ---------- ---------- ---------- ---------- [545]

Anguilla_AR-B .F.VEFF... .R........ .......... .......... ...-----.. [590]

Heterotis_AR-A ---------- ---------- ---------- ---------- ---------- [545]

Anguilla_AR-A .F.MEFF... .R.....A.. .......... .......... ...-----.. [590]

Salmo_AR-A ---------- ---------- ---------- -----....G ...-----.. [555]

Oncorhynchus_AR-A1 MY.MEFF... .R.....AE. .......... .......... ...-----.. [590]

Oncorhynchus_AR-A2 MF.MEFF... .R.....A.. .......... .......... ...-----.. [590]

Carassius_AR-A IF.MEFFL.. .R.....S.. .......... .......... ...-----.. [590]

Labeo_AR-A ---------- ---------- ---------- ---------- ---------- [545]

Danio_AR-A IF.MEFFL.. .R.....S.. .......... .......... ...-----.. [590]

Gymnocorymbus_AR-A ---------- ---------- ---------- ---------- ---------- [545]

Clarias_AR-A ---------- ---------- ---------- ---------- ---------- [545]

Oreochromis_AR-A MF.MEFF... .R.....S.. .......... .......... ...-----.. [590]

Haplochromis_AR-A MF.MEFF... .R.....S.. .......... .......... ...-----.. [590]

Oryzias_AR-A MF.MEFF... .R.....S.. .......... .......... ...-----.. [590]

Rivulus_AR-A MF.MEFF... .R.....S.. .......... .......... ...-----.. [590]

Gambusia_AR-A MF.MEFF... .R.....S.. .......... .......... ...-----.. [590]

Lepomis_AR-A ---------- ---------- ---------- ---------- ---------- [545]

Halichoeres_AR-A MF.MEFF... .RM....S.. .......... .......... ...-----.. [590]

Gasterosteus_AR-A MF.MEFF... .RM....S.. .......... .......... ...-----.. [590]

Perca_AR-A ---------- ---------- ---------- ---------- ---------- [545]

Takifugu_AR-A TY.MEFF... .R.....S.. .......... .......... ...-----.. [590]

Tetraodon_AR-A TY.MEFF... .R.....S.. .......... .......... ...-----.. [590]

Dicentrarchus_AR-A MF.MEFF... .RM....S.. .......... .......... ...-----.. [590]

Micropogonias_AR-A MF.MEFF... .RM....S.. .......... .......... ...-----.. [590]

Acanthopagrus_AR-A MF.MEFF..A .RM.M..S.. .......... .......... ...-----.. [590]

Pagrus_AR-A MF.MEFF..A .RM....S.. .......... .......... ...-----.. [590]

Pimephales_AR-A IF.MEFFL.. .R.....S.. .......... .......... ...-----.. [590]

Douard et al., Additional File 1

591

Homo_AR QKYLCASRND CTIDKFRRKN CPSCRLRKCY EAGMTLGARK LKKLGNLKLQ [640]

Gallus_AR .......... .......... .......... .......... .....S..T. [640]

Xenopus_t_AR .......... .......... .......... .......... ........A. [640]

Leucoraja_AR H.F.R..... .....V.... .........F A......G.. ..NTRPFQTA [613]

Squalus_AR H.F....... .....V.... .........F A......G.. ..NTRPFQTA [645]

Ginglymostoma_AR ---------- ---------- ---------- ----...G.. ..NTRPFQTA [561]

Protopterus_AR ---------- ---------- ---------- ----...... .....H..I. [561]

Acipenser_AR ---------- ---------- ---------- ----...... ...M---.GA [558]

Takifugu_AR-B .NH....... .....L.... .A....KR.F MS..S.KGER ---S..NRAV [626]

Tetraodon_f_AR-B ---------- ---------- ---------- -------G.R ..GAAH..NG [558]

Myoxocephalus_AR-B ---------- ---------- ---------- ----S.KG.R ..GT.QPRSG [561]

Pomatoschistus_AR-B ---------- ---------- ---------- ----S.KG.R ..GV.QARNG [561]

Oreochromis_AR-B .NH....... .....L.... .A....KR.F MS..S.KG.R ..GA.QARNG [639]

Haplochromis_AR-B .NH....... .....L.... .A....KR.F MS..S.KG.R ..GT.QASNG [639]

Gasterosteus_AR-B .NH....... .....L.... .A....KR.F MS..S.KG.R ..GN.QTRSA [639]

Oryzias_AR-B .NH....... .....L.... .A....KR.F MS..S.KG.R ..GA.QTRNG [639]

Gambusia_AR-B .NH....... .....L.... .A....KR.F MS..S.KG.R ..GA.QTRGG [639]

Heterotis_AR-B ---------- ---------- ---------- -----.EG.. M..M.Q.RPL [561]

Anguilla_AR-B .......I.. .....L.... ......KR.F A......... ...I.QMRAP [640]

Heterotis_AR-A ---------- ---------- ---------- ----...... ...I.Q..VP [561]

Anguilla_AR-A .R........ .....L.... ......K..F .......... ...I.Q..PP [640]

Salmo_AR-A .......... .....L.... .........F .......... ...I.Q..SP [605]

Oncorhynchus_AR-A1 .......... .....L.... .......R.F .......T.. ...IVQ..SP [640]

Oncorhynchus_AR-A2 .......... .....L.... .........F .......... ...I.Q..SP [640]

Carassius_AR-A .......... .....L.... .........F .......... .R.I.QM.GP [640]

Labeo_AR-A ---------- ---------- ---------- ----...... .R.I.QM.GP [561]

Danio_AR-A .......... .....L.... ......K..F .V........ .R.I.QM.GP [640]

Gymnocorymbus_AR-A ---------- ---------- ---------- ----...T.. ...IVQ..SP [561]

Clarias_AR-A ---------- ---------- ---------- ----...... ...I.Q..AP [561]

Oreochromis_AR-A .......K.. .....L.... ......K..F .......... ...I.QQ.SP [640]

Haplochromis_AR-A .......K.. .....L.... ......K..F .......... ...I.QQ.SP [640]

Oryzias_AR-A .......K.. .....L.... ......K..F .......... ...I.QQ.TS [640]

Rivulus_AR-A .......K.. .....L.... ......K..F .......... ...I.QQ.NP [640]

Gambusia_AR-A .......K.. .....L.... ......K..F .......... ...I.QQ.NP [640]

Lepomis_AR-A ---------- ---------- ---------- ----...... ...I.QQ.NP [561]

Halichoeres_AR-A .......K.. .....L.... ......K..F .......... ...I.QQ.NP [640]

Gasterosteus_AR-A .......K.. .....L.... ......K..F .......... ...I.QQ.IP [640]

Perca_AR-A ---------- ---------- ---------- ----...... ...I.QQ.NP [561]

Takifugu_AR-A .......K.. .....L.... .........F .......... ...I.QQ.NP [640]

Tetraodon_AR-A .......K.. .....L.... .........F .......... ...I.QQ.NP [640]

Dicentrarchus_AR-A .......K.. .....L.... .........F .......... ...I.QQ.NL [640]

Micropogonias_AR-A .......K.. .....L.... .........F ......R... ...I.QQ.NP [640]

Acanthopagrus_AR-A .......K.. .....L.... .........F .......... ...I.QQ.NP [640]

Pagrus_AR-A .......K.. .....L.... .........F .......... ...I.QH.NS [640]

Pimephales_AR-A .......... .....L.... .......R.F .......... .R.I.QV.GP [640]

Douard et al., Additional File 1

641

Homo_AR EEGE------ ---------- -------ASS TTSPTEETTQ KLTVSHIEGY [667]

Gallus_AR D.A.------ ---------- -------.A. SS.....QAP .MVMT.VN.F [667]

Xenopus_t_AR ..L.------ ---------- ------GSPV QGEGSKDLAP GMGIPQL... [668]

Leucoraja_AR ..TD------ ---------- ----SSVTQK QQDTSLVIVP RIGIPRMQKF [643]

Squalus_AR ..TD------ ---------- ----SSVIQK QQDT.LSIVP RIG.PRMQKF [675]

Ginglymostoma_AR .DTD------ ---------- ----SSVVQK QQNT.VSIVP RIG.PRMQKF [591]

Protopterus_AR ..S.------ ---------- ----SGSGMN QQELVPQGSA ESGPPLLDQF [591]

Acipenser_AR .DIG------ ---------- ------NQPQ NNAMQNAAM. QIDIVRM.VF [586]

Takifugu_AR-B S.-------- ---------- ------PG.A ASR.HS--S. T.A..IPPAL [650]

Tetraodon_f_AR-B ..EHLLPAWR SGELGERAGN KDGVLEPGNG -------VSR A.V.AIPPAL [601]

Myoxocephalus_AR-B ..EQ-----G PG-------K RDVLLEPG.L VVRAP--.S. D.AFCIPPTV [597]

Pomatoschistus_AR-B ..DQ-TGAAQ WGERAERAGR KD--LEPG.. AA.RPQNGS- SMGI--PPSL [605]

Oreochromis_AR-B ..EQQPASWG QGEKEERAAK KDVVLESGNA GVRAQGAAS. A.GAAIPPPL [689]

Haplochromis_AR-B ..EQQPASWG HGEKEERAAK KDAVLESRNA AVRAQG-AS. A.VAAIPPPL [688]

Gasterosteus_AR-B ...S------ -----GERAG KRDVMEPG.A AVMAQTSQAL A.GI--PPTL [676]

Oryzias_AR-B ..EQQPTAWG PGEREER--K KDVIPEPGNM ASTAQGTGP. G.PPGIPPTL [687]

Gambusia_AR-B ..EQQPGAWG HGEREG---K HNVVLEPGNA AARAQG--P. L.GI--PPTM [682]

Heterotis_AR-B R.A------- ---------- ------GSHG FAPGELGISP .SGP----SL [584]

Anguilla_AR-B .D.Q------ ---------- -------GQG PAEAELSVSP .YDL----.F [663]

Heterotis_AR-A ..S------- ---------- ------PPQ. SSDAIQTVSP .SVI----SF [584]

Anguilla_AR-A D.L------- ---------- ------PTQA P.DAIQCISP .PGL----SF [663]

Salmo_AR-A ..DL------ ---------- ------PTQG P.DAIQCISP QSGL----TF [629]

Oncorhynchus_AR-A1 ..DL------ ---------- ------PTQG P.DTIQGVSP QSGL----SF [664]

Oncorhynchus_AR-A2 ..DL------ ---------- ------PTQG P.DAIQCVSP QSGL----TF [664]

Carassius_AR-A ..VG------ ---------- ------PVQG PSETIQCLSP .PSL----TF [664]

Labeo_AR-A ..VG------ ---------- ------PSHG PSEAVQCLSP .PSL----TF [585]

Danio_AR-A D.VG------ ---------- ------AVQG PSETVQCLSP .PNL----TF [664]

Gymnocorymbus_AR-A ..DL------ ---------- ------PTQG P.DTIQGVSP QSGL----SF [585]

Clarias_AR-A ..EGLASA-- ---------- ----MPSG.G SVELIRNPSP .SG.----NF [591]

Oreochromis_AR-A K.DH------ ---------- ------G.QD PVEVIPNVSP .TG.----QL [664]

Haplochromis_AR-A ..EH------ ---------- ------G.QD PVD.H--VSP .SG.----QL [662]

Oryzias_AR-A .DDL------ ---------- ------PVQE PAELPHH.SP QSGP----NF [664]

Rivulus_AR-A ..DH------ ---------- ------SVHE PADVPHNISP .AGP----NF [664]

Gambusia_AR-A ..EH------ ---------- ------PGQE APEVPHNMFP .SGP----SL [664]

Lepomis_AR-A .DDH------ ---------- ------PGQD PLEAIQNVSP .SGL----NF [585]

Halichoeres_AR-A ..DH------ ---------- ------PGQE QPDAMQNISP .SGL----NF [664]

Gasterosteus_AR-A ..EP------ ---------- ------PLKD PLEVIQNFSP .SGL----NF [664]

Perca_AR-A ..DH------ ---------- ------PLQD PSEVIQNFSP .SGL----NF [585]

Takifugu_AR-A ..DH------ ---------- ------LVQD -LEVIHNASP .SGL----TF [663]

Tetraodon_AR-A ..DH------ ---------- ------LVQD -LEVIQNVSP .SGL----TF [663]

Dicentrarchus_AR-A ..DH------ ---------- ------PIQE PVEVIQNISP .SGL----NF [664]

Micropogonias_AR-A ..DH------ ---------- ------SVQD PSEVMQNISP .SGL----NF [664]

Acanthopagrus_AR-A DGDH------ ---------- ------PPQE PAEVMPNISP .TGL----SF [664]

Pagrus_AR-A D.DH------ ---------- ------PLQE PAEVMPNISP .SGL----SF [664]

Pimephales_AR-A D.VG------ ---------- ------SVQG PSESAQCLSP .PCL----TF [664]

Douard et al., Additional File 1

668

Homo_AR ECQPIFLNVL EAIEPGVVCA GHDNNQPDSF AALLSSLNEL GERQLVHVVK [717]

Gallus_AR .......... .....A.... ....S..... SN........ ......Y... [717]

Xenopus_t_AR S......... .....V.... .......... .L........ .......... [718]

Leucoraja_AR Q...L..A.. QS...DM.Y. .Y..T...TS .S..T..... ......R... [693]

Squalus_AR Q...L..A.. QS...DM.Y. .Y..T...TS .S..T..... ......R... [725]

Ginglymostoma_AR Q...L..A.. QS...DM.Y. .Y..T...TS .S..T..... ......R... [641]

Protopterus_AR Q...A...I. .....IT.YS ....SL..TA .N..T..... .......... [641]

Acipenser_AR QN..V..... .....DT.F. ....GL...S .N..T..... ....M.LM.. [636]

Takifugu_AR-B SSCLSL.S.. QT...AL.N. .Y.HS....P TS..T..... ......S..R [700]

Tetraodon_f_AR-B SSFHSL.S.. QT...T..N. ...HS....P TL..T..... ......S..R [651]

Myoxocephalus_AR-B RSCLSL.S.. Q....A..N. ...HA....P VS..T..... ......T..R [647]

Pomatoschistus_AR-B RSRLSL.T.. QS...A..N. ....A....S S...T..... ......T..R [655]

Oreochromis_AR-B HSCLSL.SI. Q....A..N. ...PA....P MS..T...K. ......T..R [739]

Haplochromis_AR-B HSCLSL.SI. Q....AL.N. ...PA....P VS..T..... ......T..H [738]

Gasterosteus_AR-B RSCLSL.S.. Q....A..N. ...HA....P VS..T..... ......T..R [726]

Oryzias_AR-B RSCLSL.SI. QS...TL.N. ...PA....P SS..T..... ......T..R [737]

Gambusia_AR-B RSCLSL.TI. QS...A..N. ...PA....P .S..T..... ......T..R [732]

Heterotis_AR-B S..QT..SI. .N...T..N. ..NHA....A .S........ ......E... [634]

Anguilla_AR-B HT.SM...I. .....E..N. ...YG....A .S..T..... ......K... [713]

Heterotis_AR-A QS.EV...I. .....E..N. ...HG....A .S..T..... ......K... [634]

Anguilla_AR-A PT.SV...I. .S...E..N. ...YG.T..A .T..T..... .....FK... [713]

Salmo_AR-A HS.LV...I. .S...E..N. ...HC....A .V..T..... ......K... [679]

Oncorhynchus_AR-A1 HS.LV...I. .S...E..H. ...QC....A ....T..... ......K... [714]

Oncorhynchus_AR-A2 HS.LV...I. .S...E..N. ...HC....A .V..T..... ......K... [714]

Carassius_AR-A HS.L....I. .S...E..N. ...HA....A V...T..... ......K... [714]

Labeo_AR-A HS.L....I. .....E..N. ...HA....A S...T..... ......K... [635]

Danio_AR-A HS.L....I. .....E..N. ...HG....A ....T..... ......K... [714]

Gymnocorymbus_AR-A HS.LV...I. .S......H. ...QC....A ....T..... ......K... [635]

Clarias_AR-A HS.LV..SI. .S...E..N. ...HA....A ....T..... ......K... [641]

Oreochromis_AR-A NSHLV...I. .S...E..N. ...CG....A DT..T..... ......K... [714]

Haplochromis_AR-A NSHLV...I. .S...E..N. ...YG....A .T..T..... ......K... [712]

Oryzias_AR-A NA.LV...I. .S...E..N. ...CG....A .S..T..... ......K... [714]

Rivulus_AR-A NS.LV...I. .S...E..N. ...CG....A .G..T..... ......K... [714]

Gambusia_AR-A NS.MV...I. .S...E..N. ...CG....A .G..T..... ......K... [714]

Lepomis_AR-A NS.LV...I. .S...E..N. ...YG....A .T..T..... ......K... [635]

Halichoeres_AR-A NT.MA...I. .S...E..N. ...YS....A .T..T..... ......K... [714]

Gasterosteus_AR-A NT.MV...I. .S...E..N. ...YG....A .S..T..... ......K... [714]

Perca_AR-A NS.LV...I. .S...E..N. ...YG....A .S..T..... ......K... [635]

Takifugu_AR-A NS.VV...I. .S...E..N. ...YC....A .T..T..... ......K... [713]

Tetraodon_AR-A SS.VV...I. .S...E..N. ...YC....A .T..T..... ......K... [713]

Dicentrarchus_AR-A NS.LV...I. .S...E..N. ...YG....A DT..T..... ......K... [714]

Micropogonias_AR-A NS.TV...I. .S...E..N. ...YG....A .T..T..... ......K... [714]

Acanthopagrus_AR-A NS.VV...I. .S...E.AY. ...YG....A .T..T..... ..K...K... [714]

Pagrus_AR-A NS.VV..... .S...E..N. ...YG....A .T..T..... ......K... [714]

Pimephales_AR-A HS.L....I. .....E..N. ...HA....A ....T..... ......K... [714]

* * *** * * ** *** * ** * *

Douard et al., Additional File 1

718

Homo_AR WAKALPGFRN LHVDDQMAVI QYSWMGLMVF AMGWRSFTNV NSRMLYFAPD [767]

Gallus_AR .......... .......SI. ........I. .......... .......... [767]

Xenopus_t_AR .......... ...S...T.. ........I. .......K.. .......... [768]

Leucoraja_AR ...V...... .......SL. .....AV... ......YRI. .A........ [743]

Squalus_AR ...V...... .......SL. .....AV... ......YRI. .......... [775]

Ginglymostoma_AR ...V...... .......SL. ...------- ---------- ---------- [664]

Protopterus_AR .......... .QEE.K.... ...------- ---------- ---------- [664]

Acipenser_AR ...G....Q. ...E...KI. ...------- ---------- ---------- [659]

Takifugu_AR-B ....I....D .......S.. .L....V... .L....Y.LT .CSL...... [750]

Tetraodon_f_AR-B .......--- ---------- ---------- ---------- ---------- [658]

Myoxocephalus_AR-B ....I....E M......S.. .L.------- ---------- ---------- [670]

Pomatoschistus_AR-B ....I....D ..-------- ---------- ---------- ---------- [667]

Oreochromis_AR-B ....I....D .......S.. .L....V... .L....Y.LT ..S....... [789]

Haplochromis_AR-B ....I....D .Y.....SL. .L....V... .L....Y.LT ..S....... [788]

Gasterosteus_AR-B ....I....D M......S.. .L....V... .L....Y.LT ..SL...... [776]

Oryzias_AR-B ....I....D .......S.. .L....V..L .L....Y.LT .CS....... [787]

Gambusia_AR-B ....I....D .......S.. .L....V... .L....Y.LT .CS....... [782]

Heterotis_AR-B ...GM...P. .......T.. .H.------- ---------- ---------- [657]

Anguilla_AR-B ...GM....S .Y.....T.. .H...AV... .L.....K.. K......... [763]

Heterotis_AR-A ...G...... .......TI. .HT------- ---------- ---------- [657]

Anguilla_AR-A ...G...... M......T.. .HA...V... .L....YK.. .A........ [763]

Salmo_AR-A ...GM..... ...------- ---------- ---------- ---------- [692]

Oncorhynchus_AR-A1 ...G...... .......T.. .H....V... GL....YK.A .C.L...... [764]

Oncorhynchus_AR-A2 ...GM..... .......T.. .H....V... GL....YK.. .A........ [764]

Carassius_AR-A ...G...... .......T.. .HT...V... .L....YK.A .A........ [764]

Labeo_AR-A ...G...... .......T.. .HT.------ ---------- ---------- [659]

Danio_AR-A ...G...... .......T.. .HT...M... .L....YK.A .A........ [764]

Gymnocorymbus_AR-A ...G...... .......T.. .H.------- ---------- ---------- [658]

Clarias_AR-A ...G...... .......TM. .HA------- ---------- ---------- [664]

Oreochromis_AR-A ...G...... .......TI. .Q....V... .L..K.YK.A SG.I...... [764]

Haplochromis_AR-A ...G...... .......TI. .Q....V..C .L....YK.A SG........ [762]

Oryzias_AR-A ...G...... .......T.. .Q...TV... .L....YK.. .G........ [764]

Rivulus_AR-A ...G...... .......T.. .Q....V... VLI...YK.. .G........ [764]

Gambusia_AR-A ...G...... .......T.. .Q....V... .LV...YK.. .G........ [764]

Lepomis_AR-A ...G...... .......TI. .Q.------- ---------- ---------- [658]

Halichoeres_AR-A ...G...... .....P.T.. .Q....V... .L....YK.. .......... [764]

Gasterosteus_AR-A ...G...... .......T.. .H....V... .L....YK.. .G........ [764]

Perca_AR-A ...G...... ..M....T.. .H.------- ---------- ---------- [658]

Takifugu_AR-A ...G...... .......T.. .Q....V... .L....YK.. .G........ [763]

Tetraodon_AR-A ...G...... .......T.. .Q....V... .L....YK.. .G........ [763]

Dicentrarchus_AR-A ...G...... ..M....T.. .H....V... .L....YK.A .......... [764]

Micropogonias_AR-A ...G...... .......T.. .H....V... .L....YK.. .G........ [764]

Acanthopagrus_AR-A ...G...... .......T.. ......V... GL....YK.. .G........ [764]

Pagrus_AR-A ...G...... .......T.. .H....V... GL....YK.. .G........ [764]

Pimephales_AR-A ...G...... .......T.. .HT...V... .L....YK.A .A........ [764]

*** **

Douard et al., Additional File 1

768

Homo_AR LVFNEYRMHK SRMYSQCVRM RHLSQEFGWL QITPQEFLCM KALLLFSIIP [817]

Gallus_AR .......... .......... .Q........ .......... ....F..... [817]

Xenopus_t_AR .......... .........L .......... ....E..... .......... [818]

Leucoraja_AR .....Q..Q. .T..NL.ME. Q......Q.. .LSQD..... .V........ [793]

Squalus_AR .....Q..Q. .T..NL..E. QR.....Q.. .LSQD..... .V........ [825]

Ginglymostoma_AR ---------- ---------- ---------- ---------- ---------- [664]

Protopterus_AR ---------- ---------- ---------- ---------- ---------- [664]

Acipenser_AR ---------- ---------- ---------- ---------- ---------- [659]

Takifugu_AR-B ....DK...L .S..EH.M.. KL...R.YM. KV.EE..... ...V....M. [800]

Tetraodon_f_AR-B ---------- ---------- ---------- ---------- ---------- [658]

Myoxocephalus_AR-B ---------- ---------- ---------- ---------- ---------- [670]

Pomatoschistus_AR-B ---------- ---------- ---------- ---------- ---------- [667]

Oreochromis_AR-B .I..DQ..QA .S..EH.... KL...RLCM. KV.QE..... ...V.L..M. [839]

Haplochromis_AR-B ....DQ..QV .S..EH.... KL...RLCM. KV.QE..... ...V.L..M. [838]

Gasterosteus_AR-B ....DQ..EV .S..EH.... KL...R.CM. KV.QE..... ...V....M. [826]

Oryzias_AR-B ....DQ..QI .S..EH.... KL.A.R.HR. EV.EE..... ...V....L. [837]

Gambusia_AR-B ....DQ..QV .S..EH.... KL.A.R.CK. EV.EE..... ...V....M. [832]

Heterotis_AR-B ---------- ---------- ---------- ---------- ---------- [657]

Anguilla_AR-B .....H..QV .T..EH.I.. KNF....AM. .VSQE..... .......T.. [813]

Heterotis_AR-A ---------- ---------- ---------- ---------- ---------- [657]

Anguilla_AR-A ....DR..RV .S..EH.I.. ..M....VL. .V.H...... .......... [813]

Salmo_AR-A ---------- ---------- ---------- ---------- ---------- [692]

Oncorhynchus_AR-A1 ....DH...I .S.FDH.I.. .Q.....VL. .V.QE..... .......... [814]

Oncorhynchus_AR-A2 ....DH...I .S.FEH.I.. .....Q.VL. .V.QE..... .......... [814]

Carassius_AR-A ....DH...I .S..EH..Q. K......VL. .V.QE..... .......... [814]

Labeo_AR-A ---------- ---------- ---------- ---------- ---------- [659]

Danio_AR-A ....DR...V .S..EH..Q. K......VL. .V.QE..... .......V.. [814]

Gymnocorymbus_AR-A ---------- ---------- ---------- ---------- ---------- [658]

Clarias_AR-A ---------- ---------- ---------- ---------- ---------- [664]

Oreochromis_AR-A .....H...V .T..EH.I.. .......EM. ...QE..H.. .......... [814]

Haplochromis_AR-A .....H...V .T..EH.I.. .......EM. ...QE..... .......... [812]

Oryzias_AR-A .....H...I .T..EH.I.. .......SL. ...QE..... .......... [814]

Rivulus_AR-A .....H...V .T..EH.M.. .......VL. ...QE..... .......... [814]

Gambusia_AR-A .....H..QI .T..EH.M.. .......VL. ...QE..... .......... [814]

Lepomis_AR-A ---------- ---------- ---------- ---------- ---------- [658]

Halichoeres_AR-A .....H...I .T..EH.I.. ..I....LM. ...QE..... ........L. [814]

Gasterosteus_AR-A .....H..QM .T..EH.I.. .......LL. ..SQE..... ........L. [814]

Perca_AR-A ---------- ---------- ---------- ---------- ---------- [658]

Takifugu_AR-A .....H..QL .T..EH.I.. .......VL. ..SQE..... ...I....L. [813]

Tetraodon_AR-A .....H...L .T..EH.I.. .......VL. ..SQE..... ...I....L. [813]

Dicentrarchus_AR-A .....H...I .T..EH.I.. K......LL. ...QE..... ........L. [814]

Micropogonias_AR-A .....H...I .T..EH.I.. .......QL. ...QE..... .......... [814]

Acanthopagrus_AR-A .....H...I .S..EH.I.. .......LL. ...QE..... .......... [814]

Pagrus_AR-A .....H...I .T..EH.I.. .......LL. ...QE..... .......... [814]

Pimephales_AR-A ....DR...I .S..EH.IQ. K......VL. .V.QE..... .......... [814]

Douard et al., Additional File 1

818

Homo_AR VDGLKNQKFF DELRMNYIKE LDRIIACKRK NPTSCSRRFY QLTKLLDSVQ [867]

Gallus_AR ........L. .......... .......... .......... ....V....H [867]

Xenopus_t_AR .E...D..C. .......... ...V.S...N ..A.S....F .......... [868]

Leucoraja_AR .E......Y. ....L...Q. ...V.SFQG. D..HNPQ... ........L. [843]

Squalus_AR .E......Y. ........Q. ...V.SFQG. DAPHNPQ... ........L. [875]

Ginglymostoma_AR ---------- ---------- ---------- ---------- ---------- [664]

Protopterus_AR ---------- ---------- ---------- ---------- ---------- [664]

Acipenser_AR ---------- ---------- ---------- ---------- ---------- [659]

Takifugu_AR-B .E.PRS.RC. ....TS.... ...LASHYGE --.TRRQ.LF ...Q...YL. [848]

Tetraodon_f_AR-B ---------- ---------- ---------- ---------- ---------- [658]

Myoxocephalus_AR-B ---------- ---------- ---------- ---------- ---------- [670]

Pomatoschistus_AR-B ---------- ---------- ---------- ---------- ---------- [667]

Oreochromis_AR-B EQ.P.S.HC. .K..TS.... ...LASHRGE --.TRTQ.LF ...Q...HL. [887]

Haplochromis_AR-B .Q...S.SC. .K..TS.... ...LASHHGE --.TRTQ.LF ...Q...YL. [886]

Gasterosteus_AR-B .E.....RC. ....TS.... ...LASHCGE --.TRTQ.LF ...Q....L. [874]

Oryzias_AR-B .E...S.RC. ....TS.... ...LASHRGE --.TRTQ.LF ...E...YL. [885]

Gambusia_AR-B .E..RS.RC. ....TS.... ...LASHHGE --.TRTQ.LF ...Q...YL. [880]

Heterotis_AR-B ---------- ---------- ---------- ---------- ---------- [657]

Anguilla_AR-B .E...G.N.. ....RS..N. ...LVSFRS. --S...E..Q ...R....L. [861]

Heterotis_AR-A ---------- ---------- ---------- ---------- ---------- [657]

Anguilla_AR-A .E......Y. .D..NT..N. ...L.N.S.. --.N..Q..I ...R.M..L. [861]

Salmo_AR-A ---------- ---------- ---------- ---------- ---------- [692]

Oncorhynchus_AR-A1 .....S..Y. ....LT..N. .G.V.NYG.. --SN..Q.L. ...R.M..L. [862]

Oncorhynchus_AR-A2 .....S..Y. ....LT..N. ...V.NYG.. --SN..Q... ...R.M..L. [862]

Carassius_AR-A .E...S..Y. .D..LT..N. ...L.NYG.. --.N.AM..Q ...R.M..L. [862]

Labeo_AR-A ---------- ---------- ---------- ---------- ---------- [659]

Danio_AR-A .E...S..Y. ....LT..N. ...L.NYG.. --.N.AM..Q ...R.M..L. [862]

Gymnocorymbus_AR-A ---------- ---------- ---------- ---------- ---------- [658]

Clarias_AR-A ---------- ---------- ---------- ---------- ---------- [664]

Oreochromis_AR-A .E...S..Y. ....LT..N. ...L.NFQTT --.NT.Q... ...R.M..L. [862]

Haplochromis_AR-A .E...S..Y. ....LT..N. ...L.NFQTT --.NT.Q... ...R.M..L. [860]

Oryzias_AR-A .E...S..Y. ....LT..N. .N.L.N.QMA --.N.PQ... ...R....L. [862]

Rivulus_AR-A .E...S..Y. ....LT..N. ...L.NYQMT --.N.PQ... ...R....L. [862]

Gambusia_AR-A .E...S..Y. ....LT.... ...L.NYQMT --.N.PQ... ...R....L. [862]

Lepomis_AR-A ---------- ---------- ---------- ---------- ---------- [658]

Halichoeres_AR-A .E...S..Y. ....LA..N. ...L.NNRM. --IN..Q... H..R....L. [862]

Gasterosteus_AR-A .E...S..Y. ....LT..N. ...L.SYRMA --AN.PQ... ...R....L. [862]

Perca_AR-A ---------- ---------- ---------- ---------- ---------- [658]

Takifugu_AR-A .E...S..Y. ....LT..N. ....ASYRMT --NN..Q... ...R....L. [861]

Tetraodon_AR-A .E...S..Y. ....LT..N. .....SYRMT --SN..Q... ...R....L. [861]

Dicentrarchus_AR-A .E...S..Y. ....LT..N. ...L.NYRMT --.N..Q... ...R....L. [862]

Micropogonias_AR-A .E...S..Y. ....LT..N. ...LVNYRMT --.N..Q... ...R....L. [862]

Acanthopagrus_AR-A .E...S..Y. ....LT..N. ...L.SYRMS --AN..Q... ...R....L. [862]

Pagrus_AR-A .E...S..Y. ....LT..N. ...L.NYRMN --.N..Q... ...R....L. [862]

Pimephales_AR-A .E...S..Y. ....LT..N. ...L.NYG.. --SN.AM..Q ...RMM..L. [862]

Douard et al., Additional File 1

868

Homo_AR PIAREL--HQ FTFDLLIKSH MVS--VDFPE MMAEIISVQV PKILSGKVKP [913]

Gallus_AR ...KD.--.. ........A. ...--..Y.. .......... .......... [913]

Xenopus_t_AR ......--.. .....FV.AQ ...--..... ..S....... ......R... [914]

Leucoraja_AR ITV.K.--.. .S...FVQ.Q SL.--.Q... .......A.. ....A.MA.. [889]

Squalus_AR ITV.K.--.. .....FVQ.Q SL.--.Q... .......A.. ....A.MA.. [921]

Ginglymostoma_AR ---------- ---------- ---------- ---------- ---------- [664]

Protopterus_AR ---------- ---------- ---------- ---------- ---------- [664]

Acipenser_AR ---------- ---------- ---------- ---------- ---------- [659]

Takifugu_AR-B SVTDTILP.T WSH.AS.NR- --TIN.N... .IS..V..H. ......V... [895]

Tetraodon_f_AR-B ---------- ---------- ---------- ---------- ---------- [658]

Myoxocephalus_AR-B ---------- ---------- ---------- ---------- ---------- [670]

Pomatoschistus_AR-B ---------- ---------- ---------- ---------- ---------- [667]

Oreochromis_AR-B SVV.K.--.. ..Y..F.QAQ SMH--.S... .IS..V.IH. ......M... [933]

Haplochromis_AR-B SVV.K.--.. ..Y..F.QAQ SMQMH.S... .IS..V..H. ......M... [934]

Gasterosteus_AR-B S.V.K.--.. ..Y..FLQAQ SLQTR.N... .IS..V..H. ......M... [922]

Oryzias_AR-B SVV.K.--.. ..Y..F.QAQ SLQTR.N... .IS..V..H. ......M... [933]

Gambusia_AR-B SVV.K.--.. ..Y..F.QAQ SLQMR.N... .IS..V..H. ......M... [928]

Heterotis_AR-B ---------- ---------- ---------- ---------- ---------- [657]

Anguilla_AR-B .VLKK.--.. .....FVQ.Q NL.NQ.C... .IS.....H. ....A.T... [909]

Heterotis_AR-A ---------- ---------- ---------- ---------- ---------- [657]

Anguilla_AR-A ..VKK.--.. .....FVQAQ SLHTK.N... .I......H. .R..A.MA.. [909]

Salmo_AR-A ---------- ---------- ---------- ---------- ---------- [692]

Oncorhynchus_AR-A1 .VV.K.--Q. .....FVQAQ SLPTK.N... .I......HL ....A.LA.. [910]

Oncorhynchus_AR-A2 ..V.K.--Q. .....F.QAQ SLPTK.S... .I......H. ....A.LA.. [910]

Carassius_AR-A ..V.K.--.. .....FVQAR SLPTK.S... .I........ ....A.LS.. [910]

Labeo_AR-A ---------- ---------- ---------- ---------- ---------- [659]

Danio_AR-A .VVQK.--.. .....FVQAR SLPTK.S... .I........ ..M.A.LS.. [910]

Gymnocorymbus_AR-A ---------- ---------- ---------- ---------- ---------- [658]

Clarias_AR-A ---------- ---------- ---------- ---------- ---------- [664]

Oreochromis_AR-A MTVKK.--.. .....FVQAQ SLHTK.S... .IG.....H. ....T.LA.. [910]

Haplochromis_AR-A MTVKK.--.. .....FVQAQ SLHTK.S... .IG.....H. ....A.LA.. [908]

Oryzias_AR-A M.VKK.--.. .....FVQAQ SLHTK.S... .IG.....H. ....A.LA.. [910]

Rivulus_AR-A MTV.K.--.. .....FVQAQ SLHTK.S... .IG.....R. ....A.LA.. [910]

Gambusia_AR-A MTVKK.--.. .....FVQAQ SLHTK.S... .IG.....H. ....A.LA.. [910]

Lepomis_AR-A ---------- ---------- ---------- ---------- ---------- [658]

Halichoeres_AR-A MTVKK.--.. .....FVQAQ SLPTK.S... .IG.....H. ....A.LA.. [910]

Gasterosteus_AR-A MTVKK.--.. .....FVQAQ SLPTK.S... .IG.....H. ..M.A.LA.. [910]

Perca_AR-A ---------- ---------- ---------- ---------- ---------- [658]

Takifugu_AR-A MTVKK.--.. .....FVQAQ SLPTK.S... .IG.....H. ....A.LA.. [909]

Tetraodon_AR-A MTVKK.--.. .....FVQAQ SLPTK.S... .IG.....H. ....A.LA.. [909]

Dicentrarchus_AR-A MTVKK.--.. .....FVQAQ SLPTK.S... .IG.....H. ....A.LA.. [910]

Micropogonias_AR-A MTVKK.--.. .....FVQAQ SLPTK.S... .IG.....H. ....A.LA.. [910]

Acanthopagrus_AR-A MTVKK.--.. .....FVQAQ SLPTK.S... .IG.....H. ....A.LA.. [910]

Pagrus_AR-A MTVKK.--.. .....FVQAQ SLPTK.S... .IG.....H. ....A.LA.. [910]

Pimephales_AR-A ..VQK.--.. .....FVQAR SLPTK.S... .I........ ....A.LS.. [910]

Douard et al., Additional File 1

914

Homo_AR IYFHTQ [919]

Gallus_AR ....AE [919]

Xenopus_t_AR L...SS [920]

Leucoraja_AR LL..E. [895]

Squalus_AR LL..E. [927]

Ginglymostoma_AR ------ [664]

Protopterus_AR ------ [664]

Acipenser_AR ------ [659]

Takifugu_AR-B .L..-- [899]

Tetraodon_f_AR-B ------ [658]

Myoxocephalus_AR-B ------ [670]

Pomatoschistus_AR-B ------ [667]

Oreochromis_AR-B .L..NV [939]

Haplochromis_AR-B .L..NA [940]

Gasterosteus_AR-B .L..DT [928]

Oryzias_AR-B .L..NT [939]

Gambusia_AR-B .L..NT [934]

Heterotis_AR-B ------ [657]

Anguilla_AR-B .L..K- [914]

Heterotis_AR-A ------ [657]

Anguilla_AR-A .L..N- [914]

Salmo_AR-A ------ [692]

Oncorhynchus_AR-A1 .L..K- [915]

Oncorhynchus_AR-A2 .L..K- [915]

Carassius_AR-A .L..K- [915]

Labeo_AR-A ------ [659]

Danio_AR-A .L..K- [915]

Gymnocorymbus_AR-A ------ [658]

Clarias_AR-A ------ [664]

Oreochromis_AR-A .L..K- [915]

Haplochromis_AR-A .L..K- [913]

Oryzias_AR-A .L..K- [915]

Rivulus_AR-A .L..K- [915]

Gambusia_AR-A .L..K- [915]

Lepomis_AR-A ------ [658]

Halichoeres_AR-A .L..K- [915]

Gasterosteus_AR-A .L..E- [915]

Perca_AR-A ------ [658]

Takifugu_AR-A .L..-- [913]

Tetraodon_AR-A .L..-- [913]

Dicentrarchus_AR-A .L..E- [915]

Micropogonias_AR-A .L..E- [915]

Acanthopagrus_AR-A .L..D- [915]

Pagrus_AR-A .L..E- [915]

Pimephales_AR-A .L..K- [915]

Douard et al., Additional File 1
