# Supplementary figures and images for: The fate of the duplicated androgen receptor in fishes: a late neofunctionalization event?
Source: BMC Evol Biol. 2008 Dec 18;8:336. doi: 10.1186/1471-2148-8-336 (PMC2637867; doi:10.1186/1471-2148-8-336)

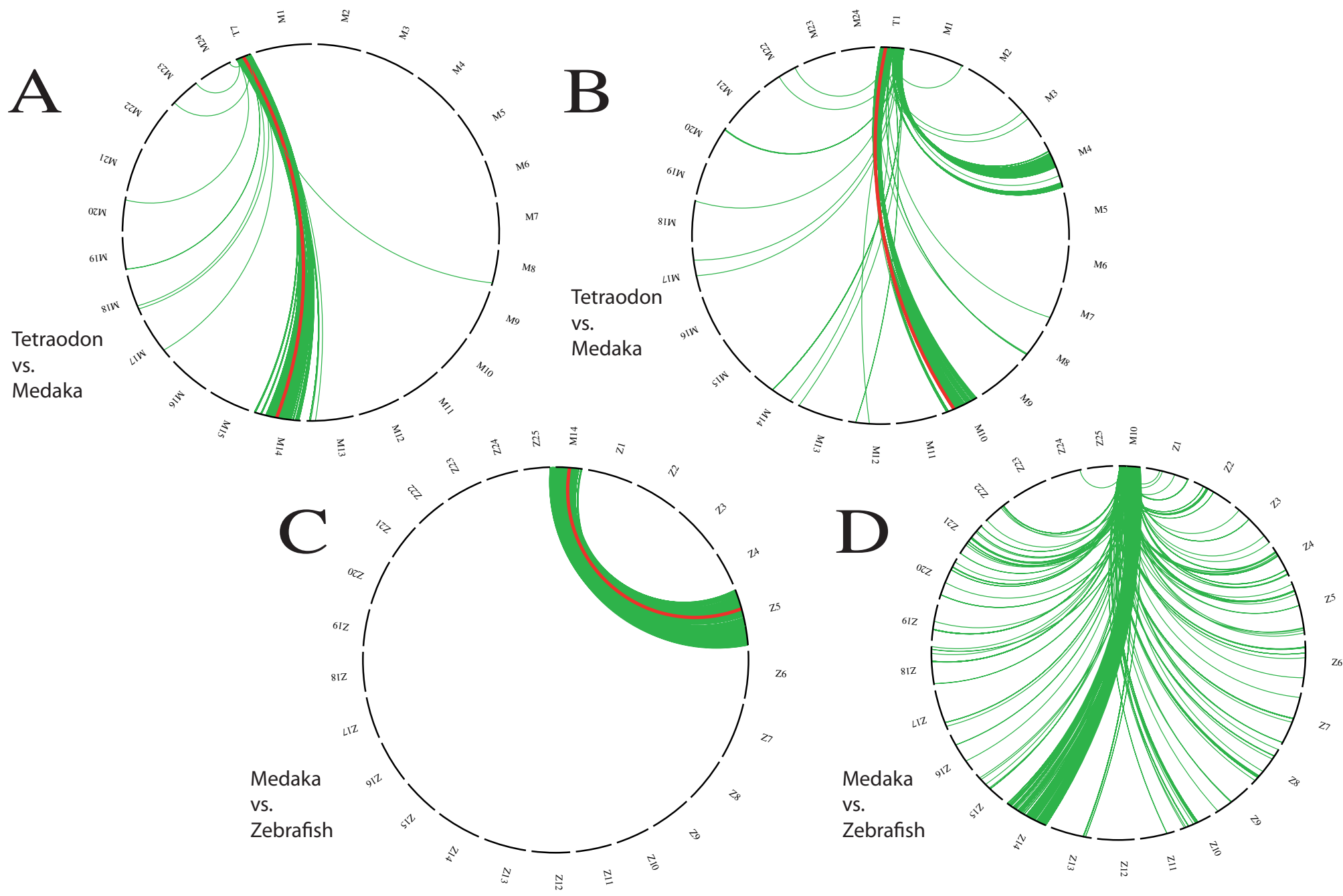

Supplement: Additional file 3 — Orthology relationships between medaka, tetraodon and zebrafish chromosomes. As referenced in legend Figure 4, these rose windows show the orthology relationship between chromosomes on which AR A and AR-B are located in the medaka, Tetraodon and the zebrafish. The excellent synteny observed between the chromosomes are strong remnants of the WGD that occurred specifically in the Teleost lineage. The red lines show the orthology link of the ARs among all the other orthologs (orthology based from Ensembl v48, in agreement with reciprocal best-hit analyses we performed, data not shown) shown here in green. (A) AR-A is found on chromosome T7 in the Tetraodon and on chromosome M14 in the medaka. A strong synteny is observed between these two chromosomes. (B) AR-B is found on chromosome T1 in the Tetraodon and on chromosome M10 in the medaka. A strong synteny is observed is also observed between these two chromosomes. (C) AR-A is found on chromosome Z5 in the zebrafish and on chromosome M14 in the medaka. An unequivocal synteny is observed between these two chromosomes, as shown in Figure 4-B between this zebrafish chromosome and that of the Tetraodon. (D) Although AR-B is found on chromosome M10 in the medaka and that a good synteny is observed with the chromosome Z14 in zebrafish, as observed in Figure 4-C for this species with the chromosome T1 of Tetraodon, the zebrafish lacks AR-B to the point we could not detect its pseudogene. (E) Table indicating the positions and Ensembl accession numbers of the relevant AR genes. [file 1471-2148-8-336-S3.pdf]

## Distribution of Inertia Percentage Between Classes and Observed Value

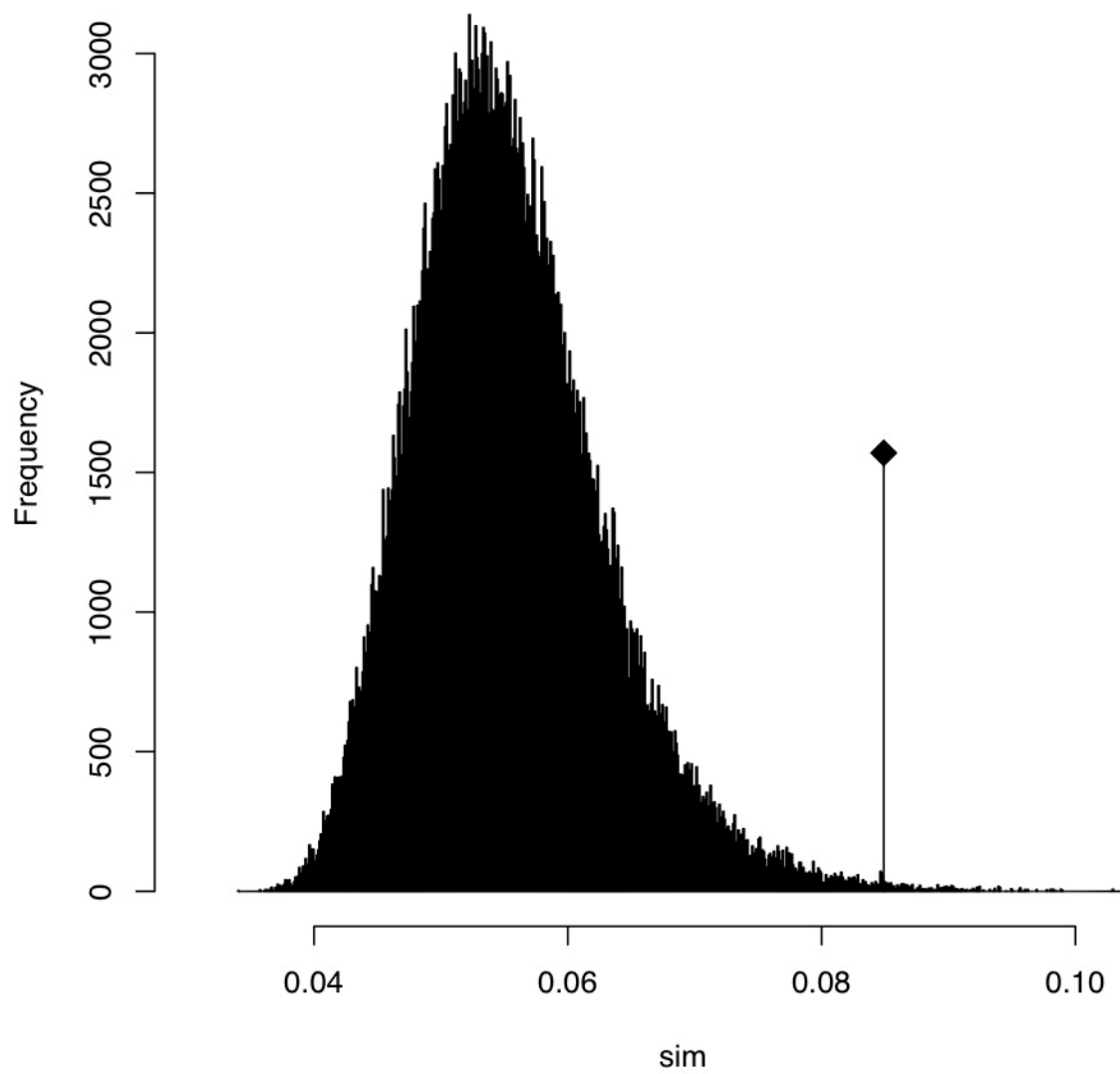

Supplement: Additional file 4 — Statistical distributions of the AR-A and AR-B substitutions in fish. Statistical representation of the distributions of the AR-A and AR-B substitutions in fish. The bell distribution is a random distribution of the substitutions, the diamond shows that the specificity of AR-A and AR-B versus the other AR is statistically clearly not random. [file 1471-2148-8-336-S4.pdf]

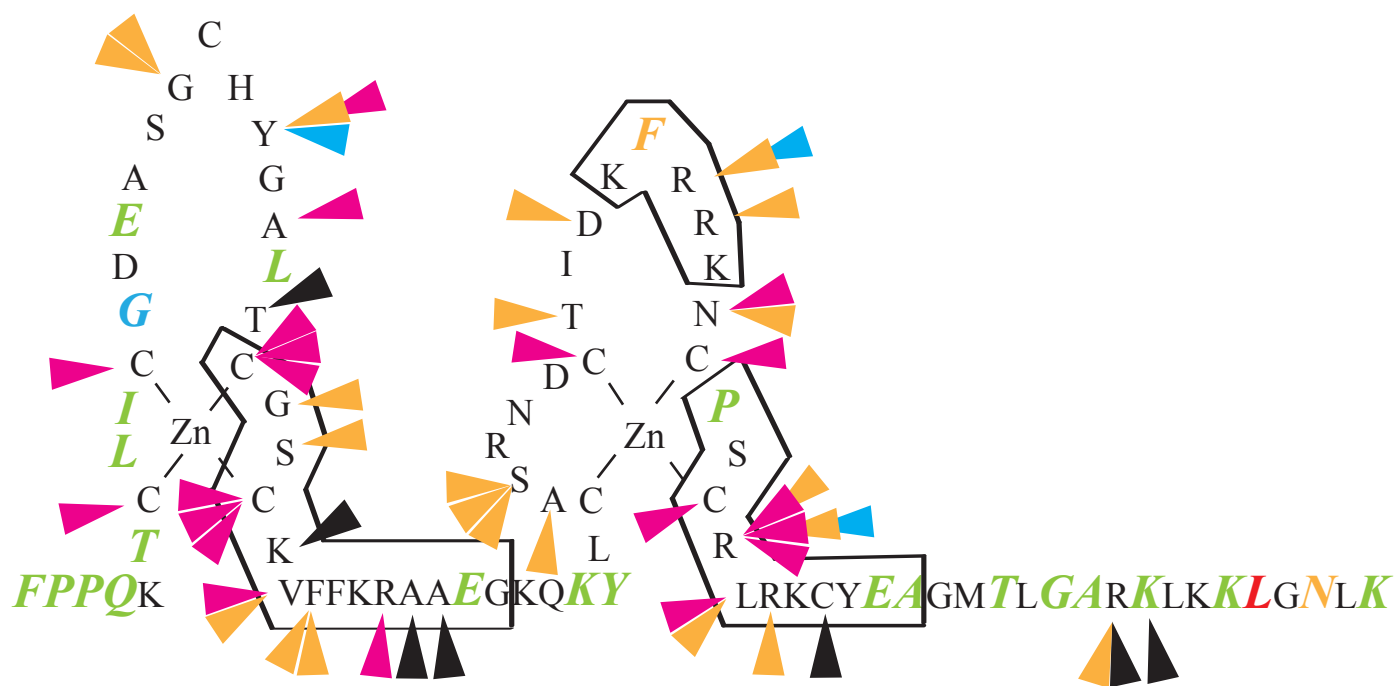

Supplement: Additional file 5 — Human and fish substitutions along the DBD of the human AR. Representation of the DBD of the human AR modified from [77]. The aa in green are the ones specific to AR-B; in blue, the ones specific to AR-A; in orange and red, when respectively the aa is hit by a common substitution or a different one when compared to other vertebrate ARs. Arrowheads refer to mutations found in CAIS, PAIS, MAIS and prostate cancer respectively in color pink, orange, blue and black. Different aa substitutions are shown by arrowheads side by side. [file 1471-2148-8-336-S5.pdf]
