# Supplementary material for: The fate of the duplicated androgen receptor in fishes: a late neofunctionalization event?
Source: BMC Evol Biol. 2008 Dec 18;8:336. doi: 10.1186/1471-2148-8-336 (PMC2637867; doi:10.1186/1471-2148-8-336)
Supplement: Additional file 2 — Phylogenetic analyses of complete AR-A and AR-B. Phylogenetic analyses of complete AR-A and AR-B with sequences encompassing the DBD and the LBD. Four methods were used: bayesian with MrBayes, maximum likelihood (ML) with PhyML with 1000 bootstrap replicates; maximum parcimony (MP) and neighbor joining (NJ) as implemented in Seaview. [file 1471-2148-8-336-S2.pdf]

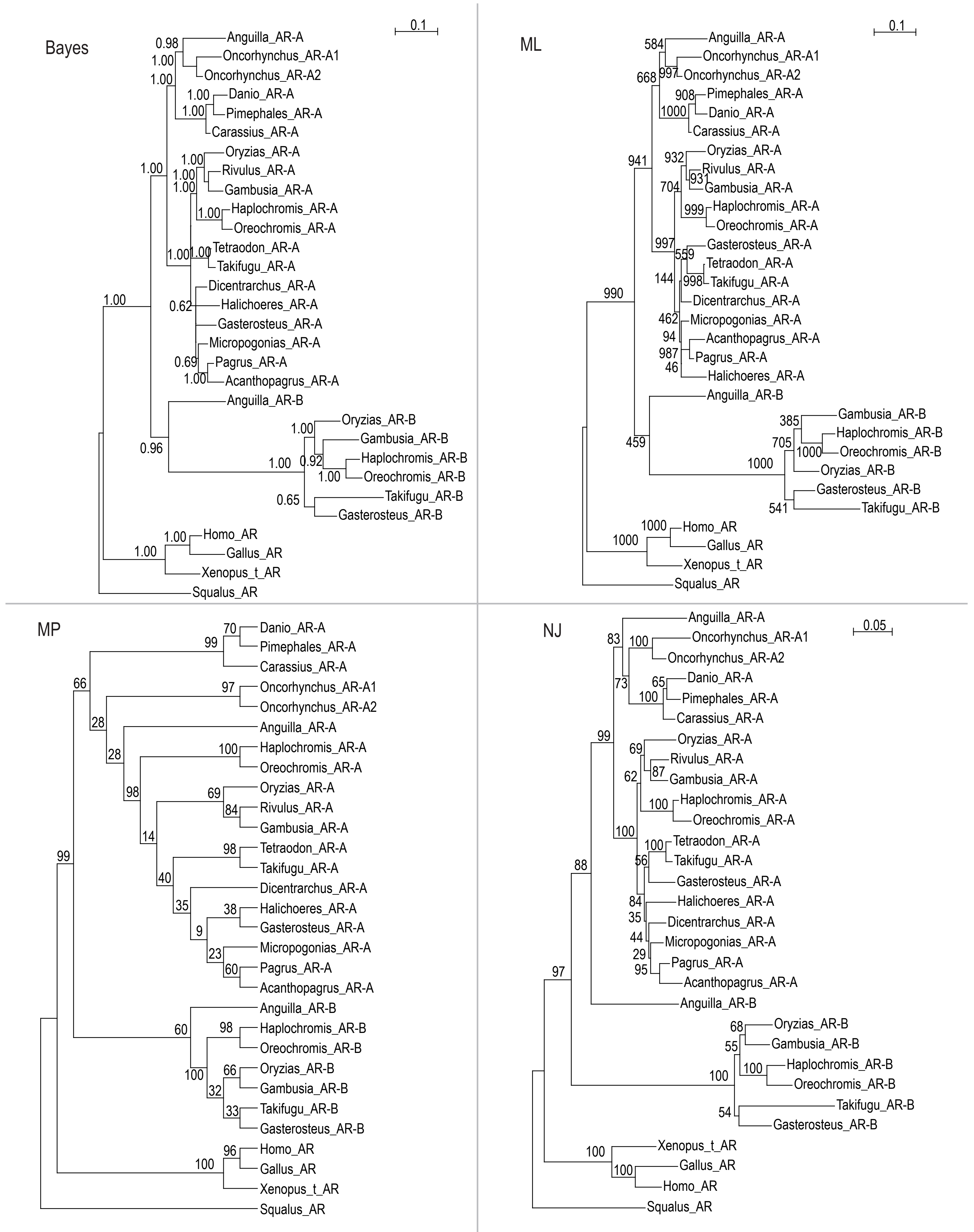

Douard et al., Additional file 2
